# Graphene-Based Transparent Flexible Strain Gauges with Tunable Sensitivity and Strain Range

**DOI:** 10.1021/acsanm.3c03967

**Published:** 2023-11-22

**Authors:** Joseph Neilson, Pietro Cataldi, Brian Derby

**Affiliations:** † Department of Materials, University of Manchester, Oxford Road, Manchester M13 9PL, U.K.; ‡ Department of Physics, Trinity College Dublin, Dublin 2, Ireland; § Smart Materials, Istituto Italiano di Tecnologia, Via Morego 30, Genova 16163, Italy

**Keywords:** reduced graphene oxide, strain gauges, piezoresistive, tunability, kirigami, channel cracks, crack patterns

## Abstract

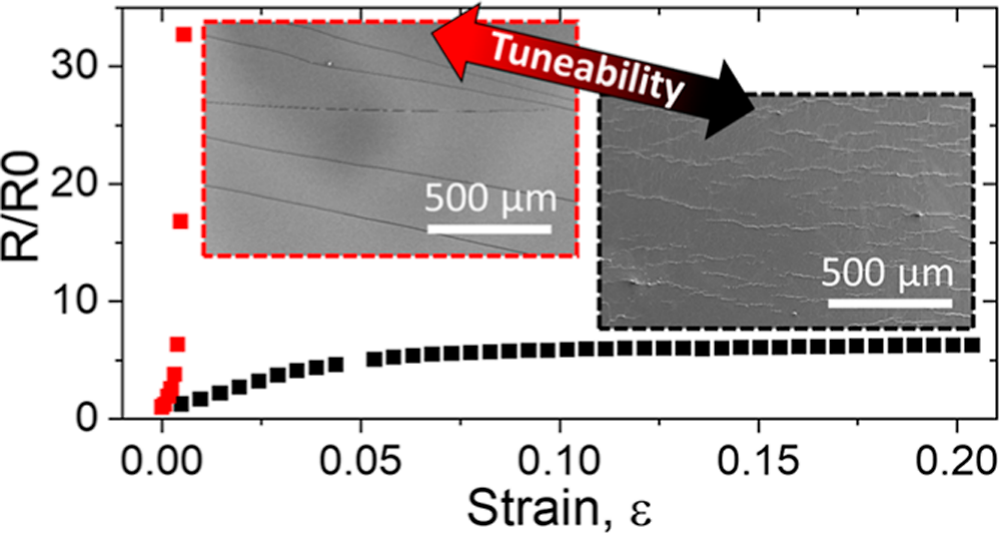

Monolayers of graphene oxide, assembled into densely packed sheets at an immiscible hexane/water interface, form transparent conducting films on polydimethylsiloxane membranes after reduction in hydroiodic acid (HI) vapor to reduced graphene oxide (rGO). Prestraining and relaxing the membranes introduces cracks in the rGO film. Subsequent straining opens these cracks and induces piezoresistivity, enabling their application as transparent strain gauges. The sensitivity and strain range of these gauges is controlled by the cracked film structure that is determined by the reducing conditions used in manufacture. Reduction for 30 s in HI vapor leads to an array of parallel cracks that do not individually span the membrane. These cracks do not extend on subsequent straining, leading to a gauge with a usable strain range >0.2 and gauge factor (GF) at low strains ranging from 20 to 100, depending on the prestrain applied. The GF reduces with increasing applied strain and asymptotes to about 3, for all prestrains. Reduction for 60 s leads to cracks spanning the entire membrane and an increased film resistance but a highly sensitive strain gauge, with GF ranging from 800 to 16,000. However, the usable strain range reduces to <0.01. A simple equivalent resistor model is proposed to describe the behavior of both gauge types. The gauges show a repeatable and stable response with loading frequencies >1 kHz and have been used to detect human body strains in a simple e-skin demonstration.

## Introduction

The ability to measure the elastic or plastic deformation of a given material has a range of applications, including monitoring response to transient mechanical stress and damage,^1,2^ sensing physiological activity of patients,^3,4^ and e-skins.^5^ Traditionally, a strain gauge is fabricated from a metal film, and deformation is computed from the variation in electrical resistance that occurs as its length and cross-sectional area change with strain. The sensitivity of the strain gauge is characterized by its gauge factor, GF, which relates the change in electrical resistance, *R*, to the imposed strain, ε, with
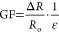
1where *R*_o_ is the electrical resistance of the unstrained gauge. Conventional metallic strain gauges typically have GF values in the range of 1–5, with working strains ε < 0.01. A number of approaches have been proposed to extend this range using new materials,^6,7^ and the principles of operation and applications of the full range of piezoresistive strain sensing mechanisms and devices have been extensively covered in recent reviews.^8^

Kang et al. demonstrated a novel, highly sensitive strain gauge,^9^ based on the change in electrical resistance of a Pt thin film deposited on a compliant polymer surface that had been previously elastically strained and relaxed, to generate a population of parallel or channel cracks, normal to the straining direction, in the conducting film. It was found that the electrical resistance of this precracked film was very sensitive to subsequent straining, with GF ≈ 1000 when ε < 0.02. They proposed that the high strain sensitivity is a consequence of the relaxed crack faces coming into partial contact once the cracking load is removed. Hence, if further deformation occurs, the crack faces begin to separate and there is a period of further separation accompanied by a proportional change (reduction) in electrical contact across the crack, which is related to the roughness of the fracture surfaces. This leads to considerable sensitivity to strain (large GF) until the cracks separate sufficiently to break the electrical contact completely. This design of strain gauge has attracted considerable interest in recent years, with various combinations of flexible substrates and conducting films proposed, which have been extensively reviewed recently.^10^

**Figure 1:**
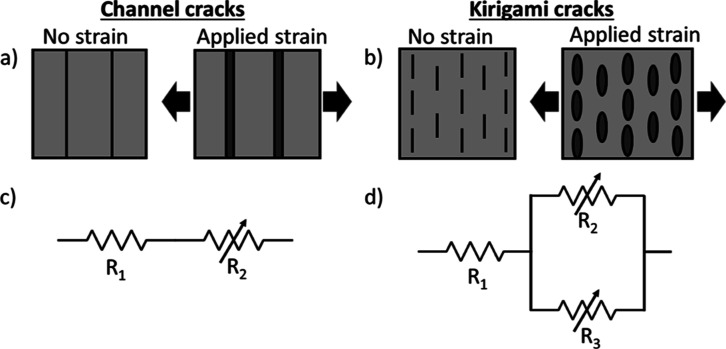
Schematic representation of crack-based strain gauge configurations: (a) channel cracks spanning the gauge width, (b) kirigami cracking with isolated cracks that do not individually span the gauge, (c) resistor model for channel cracking, and (d) resistor model for kirigami cracking.

An important distinction between these types of strain sensors is the nature of the cracking patterns induced by initial prestraining. In Kang’s initial report,^9^ the channel cracks extend across the full width of the conducting film, presenting an array of approximately parallel and evenly spaced cracks, as illustrated schematically in Figure 1a. However, under certain conditions, a different cracking scheme is observed,^11^ with repeated nucleation of cracks that grow and arrest without spanning the specimen, leading to the film being divided into an interconnected network (Figure 1b), which allows a kirigami deformation through the opening of the isolated cracks without further crack extension. In both classes of crack-based strain gauges, the usable strain measuring range is smaller than the initial prestraining, and it is believed that no further crack nucleation or growth occurs during the strain sensing procedure.

The channel crack configuration typically leads to a high GF (GF > 500) but over a relatively small strain range before the device is irreversibly damaged (ε_max_ < 0.02). In contrast, the kirigami configuration allows much larger extensions before failure (ε_max_ > 0.2) but with a lower GF (GF < 100). The electrical properties of these crack-based strain gauges can be simulated by using appropriate models of resistors in series and parallel, as shown in Figure 1. The resistance of the channel crack gauge can be adequately modeled by two resistors in series, with a constant low-value resistor, *R*_1_, representing the resistance of the uncracked film, and a much larger variable resistor, *R*_2_, representing the resistance across the cracks, which is a function of the crack opening distance and hence the applied strain normal to the crack direction. The resistance of the model gauge is thus
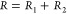
2

With kirigami cracks, there is a current path around each crack as well as one bridging the crack; to account for this, a third resistor, *R*_3_, is introduced in parallel to *R*_2_, giving the following expression for the resistance of the kirigami cracking configuration
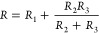
3

A more complex resistor model for *R* was proposed by Jeon et al. for kirigami cracking;^11^ however, circuit theory can be used to reduce their resistor network to the configuration displayed in Figure 1d, which we believe can be used to present a more straightforward interpretation of the piezoresistive response of kirigami-cracked films.

A number of applications for strain sensing require the sensor structure to be transparent, e.g., structural health monitoring of architectural glass and body-mounted motion sensors. Glass structures are susceptible to mechanical loading in service, and a complex laminated structure is often designed in order to improve the strength and toughness of the optically transparent structure. In safety-critical applications, e.g., windshields in high-speed rail vehicles and aircraft, there is a need to monitor load in service to sense damage evolution and schedule replacement, if required.^12−14^ In these applications the strains to be sensed will be very small, probably less than 1 millistrain (mε), and hence, a large GF is required. Here, the challenge is to introduce a strain-sensing system without compromising optical transmission through the windshield by the addition of an additional functional layer to the laminated structure. For health monitoring, or e-skin applications, which measure patient motion close to a joint, there is a different requirement of monitoring very large strains typically in the range of 0–0.5, hence the GF requirement is lower, but there must be a capability to sense over a large strain range. In these cases, transparency is a benefit because it allows for unobtrusive monitoring of patients in their normal environment without attracting unwanted attention.^15,16^ There has been some progress in this area using different sensing structures, e.g., gauges fabricated from carbon nanotube networks with an optical transmittance of 79% and a GF = 0.4 up to strains of 1.5,^17^ and gauges using multilayer graphene films with a transmittance of 75% and GF = 2.4 up to a strain of 0.018.^18^

Using the crack-based film architecture, it is possible to develop transparent strain gauges by using a suitable transparent conducting film. Such transparent, crack-based strain gauges have been fabricated using indium-doped tin oxide (ITO)^19^ and Ag nanowire networks^20^ as the conducting films with an optical transmittance of ≈0.9. The ITO film showed a channel crack morphology, leading to a maximum usable strain of ≈0.02 and a highly nonlinear piezoresistive response with GF increasing from 1 to 1000 over the measured strain range. The Ag nanowire network film showed kirigami cracking and displayed a strain range much larger than that of the ITO film gauge with GF = 60 at a strain of 1.0. These crack-based architectures present significantly larger GF values than displayed by the transparent strain gauges fabricated using different piezoresistive mechanisms that were reviewed earlier.^17,18^ Some reports demonstrate the use of graphene/ reduced graphene oxide (rGO) as a piezoresistive film material. Sakorikar et al. fabricated rGO–polydimethylsiloxane (PDMS) crack-based strain gauges with tunability of crack density and sensing range by variation of the rGO film thickness.^21^ However, these devices are not reported as transparent. Akouros and co-authors recently reported transparent strain sensors from hybrid 2D material films of rGO, fluorinated graphene, and hBN.^22^ Their strain sensing performance (GF and sensing range) can be altered by varying the ratios of each 2D material and altering the elastomeric substrate. However, the piezoresistive response and tunability mechanisms are very different from those reported here.

There are numerous ways to deposit 2D materials, such as graphene or graphene oxide (GO) onto elastomeric substrates. Conventional deposition routes such as spin coating and spray coating are widely reported but lead to stochastically deposited nanosheets. Liquid-interface assembly methods such as Langmuir–Blodgett deposition can be used to deposit GO single layers with continuously tunable packing density using the barriers of the Langmuir trough.^23^ GO nanosheets in the dispersion can be induced to form a film at the interface by injection of an “inducing agent” to destabilize the dispersion such as cationic surfactant^24^ or ethanol.^25^ The method used herein uses a liquid-interface assembly process to deposit densely tiled monolayers without the need for a Langmuir–Blodgett trough or addition of inducing agents.

Here, we present a transparent strain gauge concept based on the controlled generation of cracks in a conductive film of tiled rGO flakes deposited on an elastomeric PDMS substrate, using a 2D confined assembly at a planar liquid/liquid interface between two immiscible fluids, as described in earlier work.^26^ We show that through minor changes in the fabrication conditions, the film can be induced to form either a channel or a kirigami crack architecture. As such, we demonstrate the formation of both transparent strain gauges with a large GF value with a small measurable strain range and strain gauges with a significantly greater usable strain range but with a lower value of GF. An earlier version of this article in preprint form is available on the Arxiv Server.^27^

## Experimental Methods

GO flakes were produced using two-step electrochemical intercalation and oxidation of graphite foil, as described in detail by Cao et al.^28^ The GO nanosheets synthesized in this way are predominantly single atomic layers of mean lateral size 3.12 ± 1.27 μm. The as-received dispersion of GO in water was diluted twofold with isopropyl alcohol (IPA) to generate a 0.05 mg mL^–1^ GO ink. This was charged to a 10 mL syringe for the deposition of tiled monolayers.

**Figure 2:**
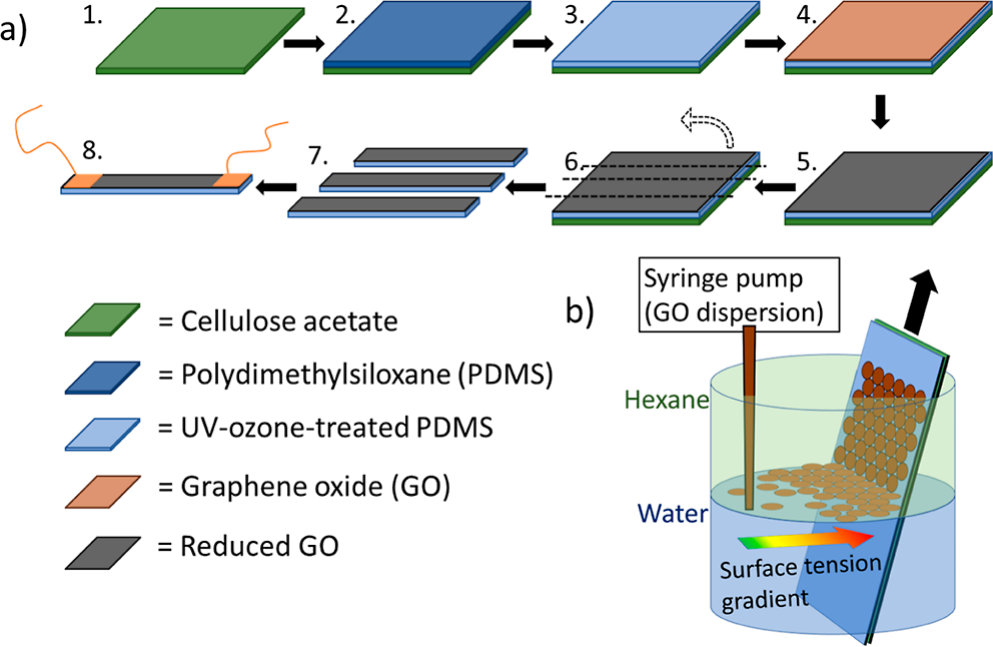
(a) Schematic workflow for the preparation of the transparent strain gauges. (b) Assembly at liquid/liquid interface and deposition onto substrate.

A schematic of the workflow used to produce the sensor structures is presented in Figure 2a. The stages of the procedure are as follows with numbering, as used in the figure.

1. Cellulose acetate films (Hartwii, Nanjing, China) were used as an A4-sized substrate for PDMS membranes.
2. PDMS membranes of Sylgard 184 two-part PDMS (Dow, Midland, MI, USA), with polymer to cross-linker ratio of 10:1 by weight, were deposited on the substrates at 500 μm thickness using an MSK-AFA-III tape caster (MTI, Richmond, CA, US) and cured at 100 °C in air for 24 h.
3. PDMS membranes were cut to manageable sizes of 3 cm × 10 cm. These PDMS substrates were treated with a ProCleaner Plus UV–ozone (UVO) plasma cleaner (Bioforce Nanosciences, Salt Lake City, UT, USA) to increase the surface energy of the PDMS surface and facilitate the deposition of GO flakes (Figure S2).
4. Deposition of GO flakes was carried out through assembly at the interface between two immiscible fluids, water and hexane (Figure 2b) (Figure S2, Supporting Information). This has been described in full detail for the assembly of MoS_2_ flakes in earlier work.^26^ Below, we present the essential details of the procedure used to produce films of GO and highlight where it differs from the procedure used to deposit MoS_2_.
5. After coating and drying, the GO flakes are reduced to rGO to increase the electrical conductivity of the film. This was achieved by placing the GO film on the PDMS substrate into a Schott bottle (500 mL). This was then placed on a hot plate at 70 °C. After 5 min, to reach thermal equilibrium, 1 or 2 drops of hydroiodic (HI) acid solution (≥57% in H_2_O, Sigma) were dropped into the bottom of the bottle. The bottle was sealed loosely with a lid, and the films were exposed to HI vapor for times up to 60 s.
6. After reduction to rGO, a blade was used to cut individual specimens of dimensions 3 cm × 1 cm.
7. The specimens were carefully removed from the cellulose acetate support.
8. For the devices used in human motion sensing, copper tape was “cold soldered” to the surface of the devices using silver conductive dispersion (186–3600, RS Pro, Northamptonshire, UK).

GO films are formed by assembly at the interface between two immiscible liquids. The full experimental details of this method and a demonstration that it leads to the formation of near-monolayer densely tiled films of 2D materials have been previously published using MoS_2_ monolayer flakes as the 2D material.^26^ A brief description of the process is as follows. GO films were confined to the interface between two immiscible fluids of different densities. Here, the denser lower phase was deionized (DI) water, density ≈1000 kg m^–3^, and the upper phase was *n*-hexane (Sigma, ≥99%), density ≈655 kg m^–3^. A conical flask with an internal neck diameter of 3 cm acted as a reservoir to contain water and hexane, with the water/hexane interface about 3 cm below the rim of the flask (Figure S2, Supporting Information). A clamp attached to a precision dip coater positioned the PDMS substrate immediately below the water surface. The GO ink in a 50:50 IPA/water suspension was charged into a syringe (10 mL, Sigma) with a 120 mm long needle (21 gauge, Sterican, VWR, Radnor, PA, USA) and inserted into a syringe pump (11 plus, Harvard Apparatus, Holliston, MA, USA). The tip of the needle was positioned at the water/hexane interface. The addition of the GO dispersion was started at 0.1 mL/min and allowed to run for 5 s before the PDMS on cellulose acetate was raised by the dip coater at a speed of 1 mm/s. The injection of the IPA/water solvent of the GO ink introduces a concentration gradient between the point of injection and the position of the substrate as it is drawn through the liquid/liquid interface (Figure 2b). This local concentration gradient establishes a consequent gradient in the interfacial tension at the hexane/water interface. This is believed to promote the dense packing of the GO flakes at the liquid/liquid interface and the subsequent transfer of a dense film onto the PDMS substrate.^26^ After the deposition process was completed, the GO film was left to dry in a fume cupboard for 1 h. The dip coating process was used to coat substrates with areas up to 25 cm^2^.

The optical transmittances of the GO and rGO films were obtained using a Lambda 25 UV–visible spectrophotometer (PerkinElmer, Waltham, MA, USA). Sheet resistance measurements were performed using a Jandel 4-point probe system (Jandel Engineering, Leighton Buzzard, UK) with 1 mm electrode spacing. A source meter (2400 series, Keithley, Cleveland, OH, USA) was used to source and measure the current and voltage of the outer and inner probe electrodes, respectively.

To allow piezoresistive characterization of the strain gauges, the devices are carefully peeled from the cellulose acetate support and placed in an in-house designed linear extension stage, with a displacement resolution of ≈1 μm. The gauge length of each specimen is defined by the spacing between adhesive copper tape electrodes (3M, Saint Paul, MN, US). The samples were then strained in the linear stage at engineering strains in the range of 0.05–1.0 to induce cracking in the conductive films. The same stage was also used to measure the change in the resistance of the cracked devices after relaxation to zero strain. The crack patterns were imaged, and crack spacings were measured using an inspection microscope fitted with a CCD camera (AxioCam ERc5s, Carl Zeiss AG, Jena, Germany). A source meter (2400 Series, Keithley) in electrical resistance measurement mode was connected to the copper tape using crocodile clips to measure the devices’ electrical resistance change under strain. For the dynamic response measurement of strain gauges, one end of the strain gauge was affixed to the cone of a loudspeaker and the other end was mounted to a fixed point using adhesive tape, such that the gauges measured the speaker cone displacement in bending mode. The dynamic response was recorded by using a Wheatstone bridge circuit connected to an oscilloscope (DSO012A, Agilent Technologies, Santa Clara, CA, USA).

## Results and Discussion

**Figure 3:**
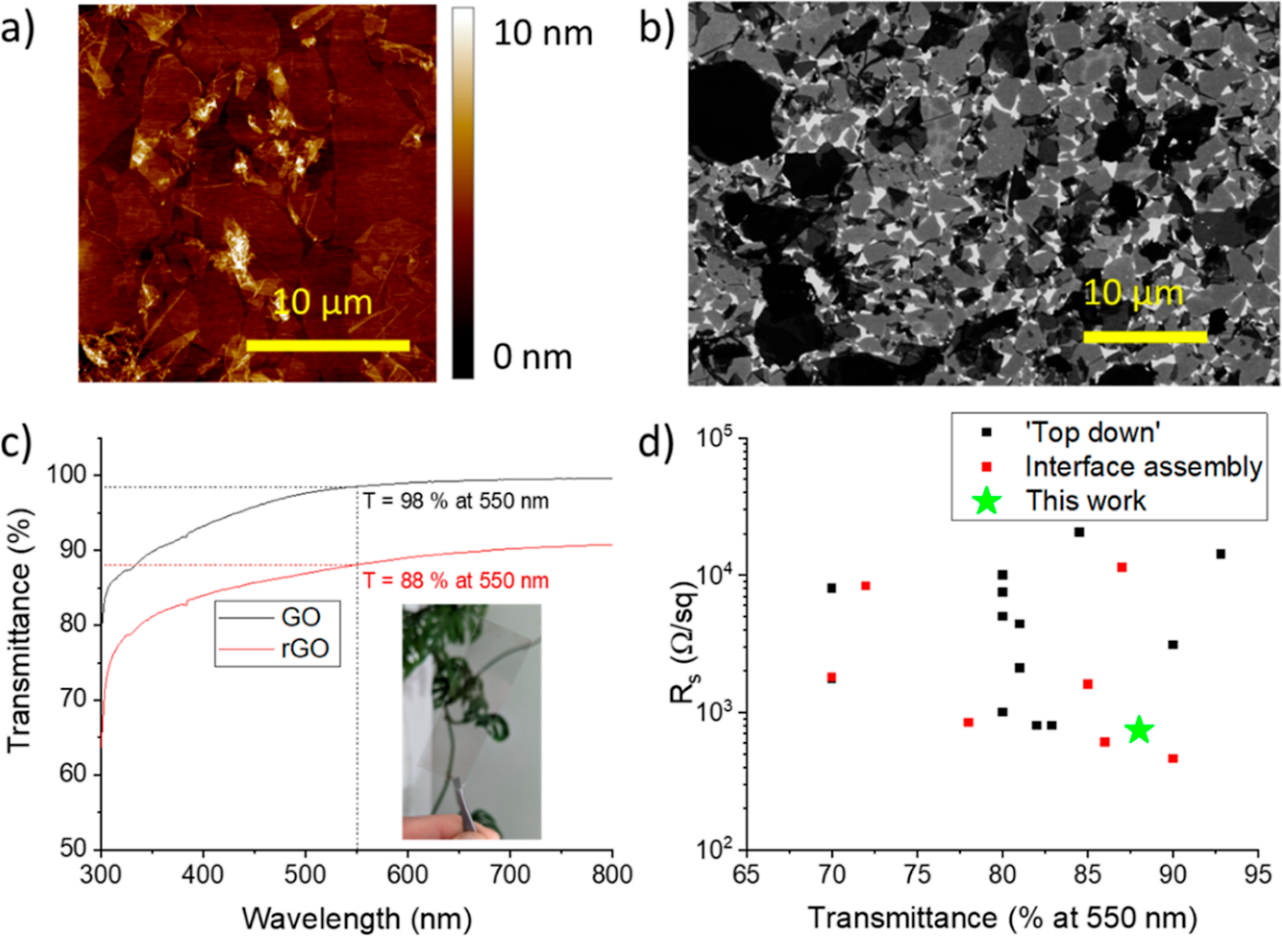
(a) AFM image of the assembled GO monolayer (height range 0–10 nm). (b) SEM image of the film after reduction to rGO. (c) Optical transparency as a function of incident wavelength for the GO/PDMS and rGO/PDMS membranes produced by assembly at the water/hexane interface. (d) Comparison of the sheet resistance and optical transparency of transparent, electrically conductive rGO films reported in the literature and this work. See Supporting Information Table S2 for full details and citation information for prior work.

Figure 3a,b shows atomic force microscopy (AFM) and scanning electron microscopy (SEM) images of the assembled GO and rGO films on the PDMS membranes. The densely packed nature of the films and the absence of significant flake overlap is evident in both images. A more detailed analysis of 2D material films (MoS_2_ monolayer and bilayer flakes) assembled using the identical method at immiscible H_2_O/hexane interfaces,^26^ confirms that such films are flatter and show significantly less variation in thickness than films produced by spin coating or spray coating. Using the contrast in SEM images as a proxy for film thickness, we have also performed a brief statistical analysis of the thickness distribution in these rGO films using the image presented in Figure 3a. The analyzed data are presented in the Supporting Information (Figure S3 and Table S1), indicating that approximately 65% of the film area shows monolayer or bilayer rGO film coverage.

After reduction of the GO films with HI vapor, the resulting rGO membrane demonstrates low sheet resistance of *R*_s_ = 850 ± 42 Ω □^–1^ and excellent transmission of 88% at 550 nm (Figure 3c,d). The optical transparency of the GO is reduced by around 10% during the reduction process, in line with the findings of other reports, indicating a restoration of the conducting π-electron system of graphene.^29,30^ Here, the optical absorbance is relatively constant over the entire visible spectrum, suggesting the good suitability of the rGO/PDMS membrane as transparent conductive electrodes (TCEs). Note that the transparency after reduction represents the change in the complete system (PDMS and rGO) rather than a change in the properties of the rGO alone. Figure 3d compares the sheet resistance and optical transmittance of our rGO/PDMS films with data from a previously published work on TCEs. Top-down (black symbols in the figure) indicates films formed by deposition techniques such as spray deposition or spin coating, while interface-assembled films (red labeled in the image) are deposited by assembly at either liquid/air (Langmuir–Blodgett) or immiscible liquid interfaces. For full details and sources of the data, refer to Table S2, Supporting Information. We believe that the high optical transmittance is the result of good areal coverage with minimal film overlap (Figure 3a,b) and reduced contact resistance through edge-to-edge flake packing.

A useful figure of merit for TCEs is the ratio of electrical-to-optical conductivity
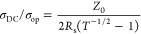
4where σ_DC_ and σ_op_ are the electrical and optical conductivity of the TCE, respectively, *Z*_0_ = 377 Ω is the impedance of free space, *R*_s_ is the sheet resistance, and *T* is the optical transmittance.^31^ The rGO TCEs presented here demonstrate an electrical/optical conductivity ratio of σ_DC_/σ_op_ = 3.36. This is within the same order of magnitude as some solution-processed ITO TCEs, further highlighting the suitability of rGO films produced at immiscible liquid interfaces for this application.^32^ Compared with previous literature examples, the rGO on PDMS films presented in this study shows one of the highest reported electrical-to-optical conductivity ratios (Supporting Information Table S2). However, we note that our rGO TCEs demonstrate the greatest conductivity ratio for rGO films fabricated at a relatively low processing temperature, <100 °C. In the literature, the greatest conductivity ratios have been reported from rGO films after annealing at over 1000 °C, which is impractical for films on polymer membranes.

### Crack Patterns in Strained rGO Films

**Figure 4:**
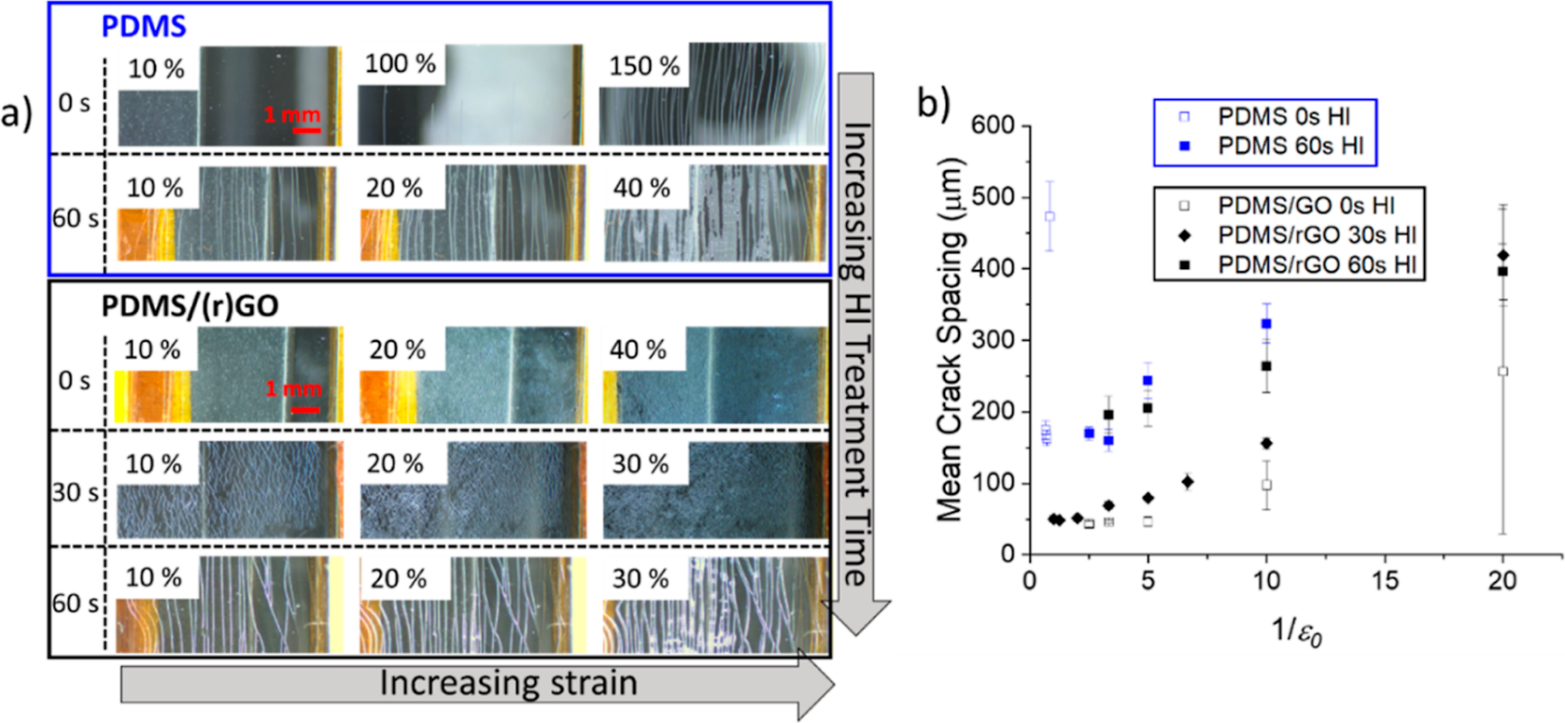
(a) Optical microscopy images illustrating the crack morphologies that form on straining PDMS samples and PDMS after the deposition of GO or rGO films. Blue-outlined images show cracks in strained PDMS films after UV–ozone surface treatment but with no deposited film. Conversely, the black-outlined images show coated films of PDMS/GO and, after a reduction of 30 or 60 s, PDMS/rGO films. In all cases, the images progress from left to right as the strain increases. Note the different strain ranges for the uncoated and coated PDMS specimens. (b) Mean crack spacing after straining as a function of the reciprocal of the applied strain for the data obtained from the images in panel (a).

By initially straining the rGO/PDMS membranes, it is possible to generate the crack structures required to sense strain.^9,10^Figure 4a shows optical micrographs of the films under various levels of strain, revealing the morphologies of the resulting crack patterns. High magnification images of the kirigami/channel crack morphologies are also given in Figure S4, Supporting Information. It is important to realize that the cracks observed are not necessarily indicative of the properties of the deposited GO or rGO film. Before depositing GO, the PDMS membranes are subjected to a UV–ozone treatment that improves the wettability of the surface for GO deposition. This treatment will also lead to a thin (<1 μm thickness) film of amorphous SiO_2_ on the surface. Such a film will be brittle, and we would expect to see cracking if the treated PDMS film is strained. This does indeed occur as is seen in the first two rows of Figure 4a (blue frame in the figure), which shows an onset of channel cracking at a tensile strain ε ≈ 1 in the UV–ozone-treated PDMS films without a GO film. These cracks nucleate and propagate rapidly across the width of the membrane, normal to the loading direction. As the strain increases, further cracks nucleate and propagate to fully span the specimen. The mean crack spacing, *h*, decreases with increasing strain to a saturation value of approximately 160 μm when ε > 1.4. If the PDMS is exposed to HI vapor for 60 s, cracking initiates at a lower strain and converges to a similar saturation value as found with the as-fabricated PDMS but at a lower strain (ε > 0.4).

The coated PDMS/GO and PDMS/rGO membranes (black frame in Figure 4a) show a different behavior. The PDMS/GO film without HI reduction shows the lowest strain at which cracking initiates. However, in this case, the cracks do not propagate across the full width of the specimen but arrest after propagating a few millimeters normal to the applied load. Further cracks nucleate parallel to the initial arrested cracks, and the mean crack spacing decreases to a saturation value of *h* ≈ 43 μm at ε > 0.2. The crack pattern is discontinuous, and as the applied strain increases, the cracks open but do not appear to extend further; this is the kirigami cracking, discussed earlier. After 30 s of exposure to HI, the new PDMS/rGO films show similar behavior to the PDMS/GO membrane, also forming a kirigami crack pattern. However, the cracks nucleate at a larger initial strain, followed by the mean crack spacing decreasing with increasing strain, in a manner similar to that seen with the PDMS/GO films but over a greater strain interval, reaching saturation at ε > 0.5. After 60 s of HI treatment, the PDMS/rGO membrane shows a transition in behavior, forming channel cracks that span the width of the membrane with the crack spacing saturating close to 160 μm.

The phenomenon of repeated nucleation of channel cracks is well-known from studies of the fracture of thin films on compliant surfaces.^33^ The solution of Thouless predicts the following relation between strain and mean crack spacing, *h*, for the simplified case when the film and substrate have the same elastic properties^34^
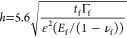
5where *E*_f_, ν_f_, Γ_f_, and *t*_f_ are Young’s modulus, Poisson’s ratio, fracture energy, and thickness of the film, respectively. More sophisticated analysis of the cracking of thin films has taken into account the effect of the mismatch in elastic properties and the stochastic nature of crack nucleation.^33,35,36^ These provide more mechanically robust formulations of the crack driving forces but at the expense of numerical solutions to the problem. These studies show that there is a significant influence of a mismatch in elastic properties on the crack growth driving force but that the general principle of Thouless’s approach is correct. Thus, we would expect the crack spacing to be inversely proportional to the maximum applied strain and this behavior is consistent with our data plotted in Figure 4b. We note that a similar relationship between crack spacing and the inverse of the applied strain is also found for the kirigami-cracked films. However, the analytical solution used to represent this relationship for channel cracking (eq 5) may not be appropriate for kirigami cracking.

### Strain Sensing Behavior

The PDMS/GO membranes show very high electrical resistance and, thus, are unsuitable for strain sensing applications. The lower electrical resistance PDMS/rGO membranes, formed after exposure to HI for 30 and 60 s, show extensive cracking patterns and have thus been tested for their suitability as strain gauges. The different reduction processes have resulted in two distinctly different crack morphologies, as presented in Figure 4, which, following the reports on the behavior of crack-based strain gauges in the literature, are expected to show different strain sensing behavior.^10^

**Figure 5:**
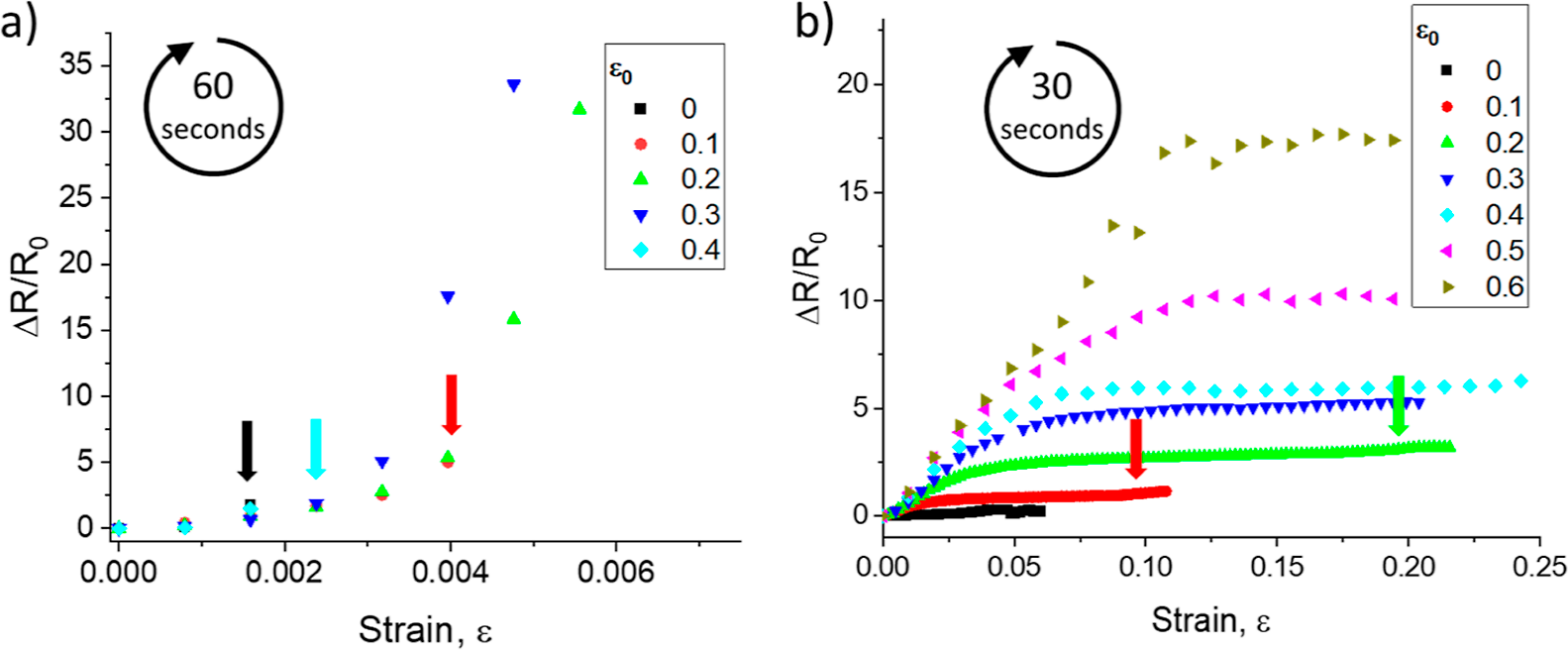
Change in resistance plotted against applied strain for rGO/PDMS membranes at different levels of conditioning strain, ε_0_. (a) Channel cracking: colored arrows indicate where gauges went open circuit during testing. (b) Kirigami cracking: colored arrows indicate a slight change in GF as the applied strain approaches the conditioning strain.

Figure 5a shows the change in resistance of the PDMS/rGO membranes exposed to HI for 60 s as a function of previously applied prestrain or conditioning strain, ε_0_, up to a maximum of ε_0_ = 0.4. In all cases, there is a region showing a linear piezoresistive response at low strains with ε < 0.003. The strain sensitivity in this regime is approximately constant with GF ≈ 800. The membranes strained to ε_0_ = 0.2 and 0.3 both show a sudden upturn in sensitivity at ε ≈ 0.003 to GFs of 16,600 and 18,000, respectively. Devices at all other conditioning strain levels failed through going open circuit at lower strains (indicated by the colored arrows in Figure 5a). The resistance of the device, after the conditioning strain is applied and the relaxation to zero strain, *R*_o_, increases rapidly with increasing ε_0_. At ε_0_ > 0.3, the devices show much greater initial resistance, with *R*_o_ close to the maximum measurable by our equipment, and hence, only a reduced strain sensing range is accessible. A complete tabulation of the performance of channel-cracked membranes subject to different conditioning strains is presented in Supporting Information Table S3. The GF of these devices at low applied strain is comparable to those reported by others for channel crack strain gauges fabricated from ITO,^19^ metal films,^9,37^ and Ag nanowires.^38^ The transition in sensitivity at high strain is similar to the report of Yang et al.^37^ for channel-cracked Au/PDMS sensors, who also found a transition to much larger GF values at strains in the range of 0.01–0.03. However, they reported that the GF in the low-strain regime decreased with an increase in conditioning strain.

Figure 5b shows the change in resistance of the PDMS/rGO membranes exposed to HI for 30 s as a function of conditioning strain up to ε_0_ = 0.6. These membranes all displayed kirigami cracking. After conditioning, the piezoresistive response is highly nonlinear, showing a large GF at low strains that gradually decreases with increasing strain to reach a stable value of GF at larger strains. The initial GF increases with conditioning strain, up to a maximum of GF = 149, at ε_0_ = 0.6. However, the GF at large strain is approximately constant, ≈3, with conditioning when ε_0_ < 0.6. This is very similar to the GF of the uncracked rGO/PDMS film (Table S4). The strain range over which the GF decreases to its stable, large strain value increases with ε_0_ but is close to 0.3 ε_0_ (Table S4). With membranes conditioned at ε_0_ > 0.6, there is a very small workable strain range and a transition to an irreversible increase in resistance occurs after relatively low strains; this is possibly associated with the formation of channel cracks. Table S3 summarizes the performance of the kirigami-cracked membranes.

The practical working strain range is limited by the conditioning strain, ε_0_. If a kirigami-cracked membrane is strained ε > ε_0_, there is a noticeable increase in the rate of change of membrane resistance (identified in Figure 5b by colored arrows). This change is believed to indicate the nucleation of further new cracks in the membrane. We also note that at strains exceeding ε = 0.2, there is some wrinkling and possible local delamination of the rGO films (Supporting Information, Figure S5). This is possibly associated with a lateral Poisson contraction of the films at large deformations of the PDMS and the rGO/silica-like layer. The performance of these devices, in terms of GF, compares well with the data from the literature for cracked strain gauges, also based on kirigami crack morphology: e.g., Luo et al., using cracked Au films with a carbon nanotube second layer, found a low strain GF = 70 that reduced to GF = 10 at ε = 0.2 and further still at greater strains;^3^ Wang et al. used carbon nanotubes as the conducting film but deposited directly onto a PDMS surface before prestraining to measure a GF = 87 at low strains and a GF = 6 at ε > 0.4.^39^ Note that neither of the kirigami network crack sensors in these earlier reports was transparent.

**Figure 6:**
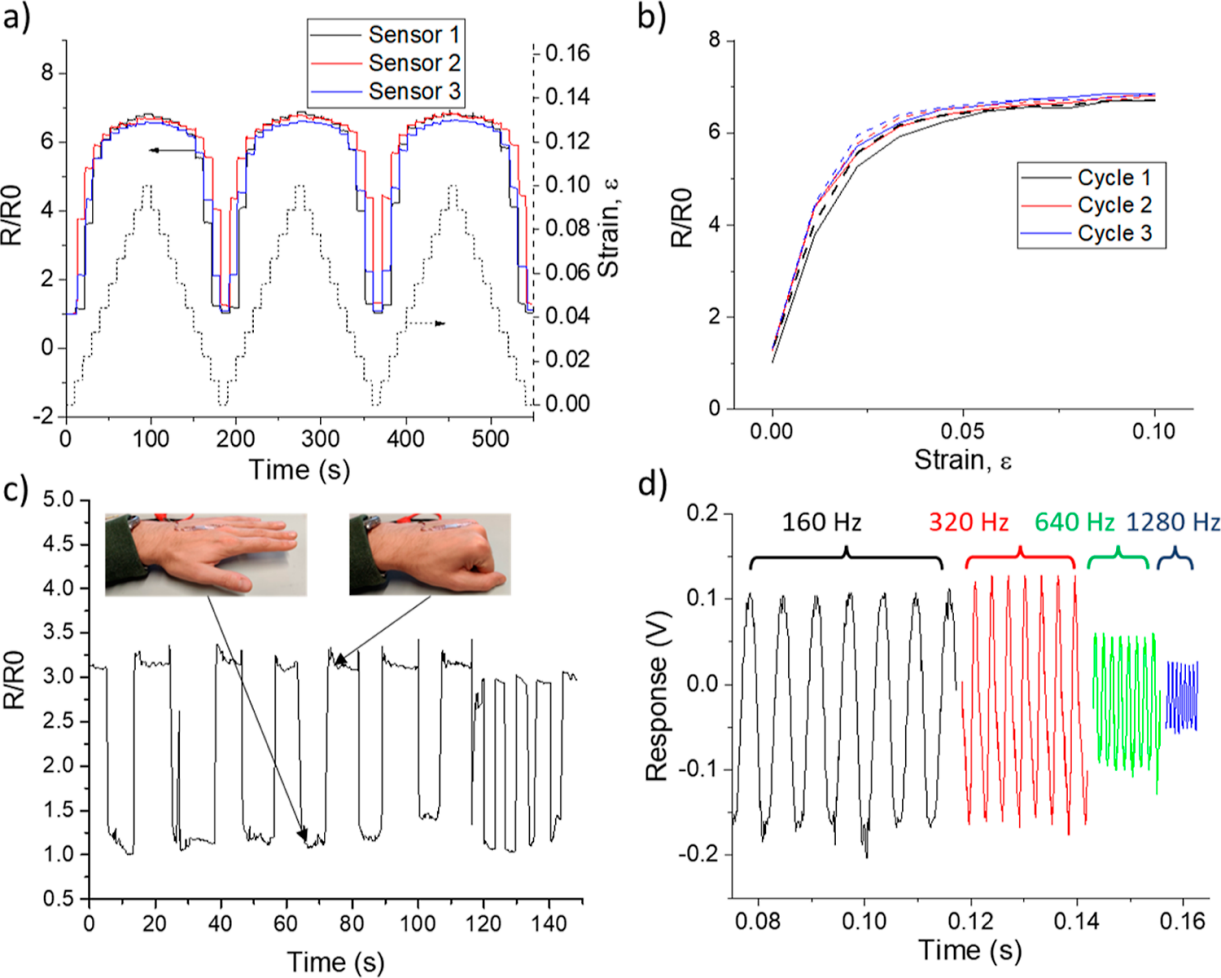
Strain sensing properties of the 30 s HI-treated kirigami-crack-based strain gauges. (a) 3× pull–release cycles performed with three different strain sensors. The data is displayed as time-dependent, and each “step” is due to applying a 100 μm extension to the sensors. The dashed line corresponds to the right *y*-axis and is the actual strain applied to the sensors. (b) Variation of resistance of a sensor extended and released for three cycles. The solid and dashed lines are the piezoresistive response during the straining and releasing stages, respectively. (c) Human motion sensing of the opening/closing of the hand by a skin-mounted strain sensor. (d) Frequency response for a typical strain sensor fixed to a loudspeaker cone.

The PDMS/rGO sensors exposed to HI for 30 s and subjected to strain conditioning of ε_0_ = 0.3 were tested for their repeatability in performance applications at high strain rates and for human motion sensing. Figure 6a shows the repeatable and reproducible piezoresistive response between three of the tested sensors, as indicated by the time-dependent response over three pull–release cycles. The sensors also demonstrate low hysteresis (Figure 6b), although there appears to be some variation in response between different cycles. Similar variation between cycles is seen in Figure 6c when the sensors were used to monitor the strain variation in a glove-mounted sensor detecting human hand motion with strain cycles in the order of 0–0.2.^40^ It is unclear whether this shows an intrinsic variability of the film or issues with the electrical connections being strained or deformed during the experiments. The strain sensors also exhibit a fast dynamic response up to 1280 Hz when mounted on an acoustic loudspeaker cone (Figure 6d). This frequency response indicates the possibility of utilizing strain sensors in applications such as human speech monitoring by mounting the devices to the neck.^9^ Furthermore, our highly transparent strain sensors would provide an invisible sensing platform for applications in the entertainment and performing arts industries such as invisible neck microphones. We also propose that the transparent gauges would be useful in healthcare applications as noninvasive and invisible skin-mounted devices.^15,16^ Furthermore, the channel crack strain sensors can be used in transparent applications such as monitoring strains in architectural, automotive, or aerospace glass components. Finally, we demonstrated the durability of these gauges by performing 6000 strain-release cycles of ε = 0.05 (Supporting Information, Figure S5). All these results provide encouraging preliminary data for applications of the devices, but further optimization of both gauge manufacture and the dynamic testing regime is required.

### Mechanisms for the Piezoresistive Effect

#### Channel Cracking

The channel crack morphology has been the most studied in prior work with other conducting films,^9,10,19,37^ and this design leads to the highest values of GF but a relatively low workable strain range. The simple series resistor model configuration in Figure 1c presents an appropriate model if the electrical resistance of a crack is considerably larger than that of the film. Table S3 presents the electrical resistance and mean crack spacing data obtained after straining the PDMS/rGO sensors that were exposed to HI reduction for 60 s with strains up to 0.3. The resistance per crack, *R*_2_*, is obtained by the following equation
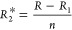
6where *R*_1_ is the resistance of the film prior to straining and *n* is the number of channel cracks in the membrane. Hence, the total resistance of the cracked membrane after the initial conditioning strain, ε_0_, is given by the modified version of eq 2
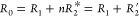
7where *R*_2_′ is the resistance after relaxation to zero strain contributed by all cracks formed during strain conditioning. From the data obtained from our conditioning strain results (Supporting Information Table S3), the relationship between crack number and conditioning strain is approximately *n* = 190ε_0_. A simple model for the increase in membrane resistance is that each crack adds a further constant value resistor in series with the contribution from the uncracked film, and thus because the number of cracks increases in direct proportion to ε_0_, we would expect the membrane resistance to also be proportional to ε_0_. However, the data in Table S3 show that the resistance increases nonlinearly with increasing conditioning strain. Thus, the resistance of each channel crack must also independently increase with strain.

**Figure 7:**
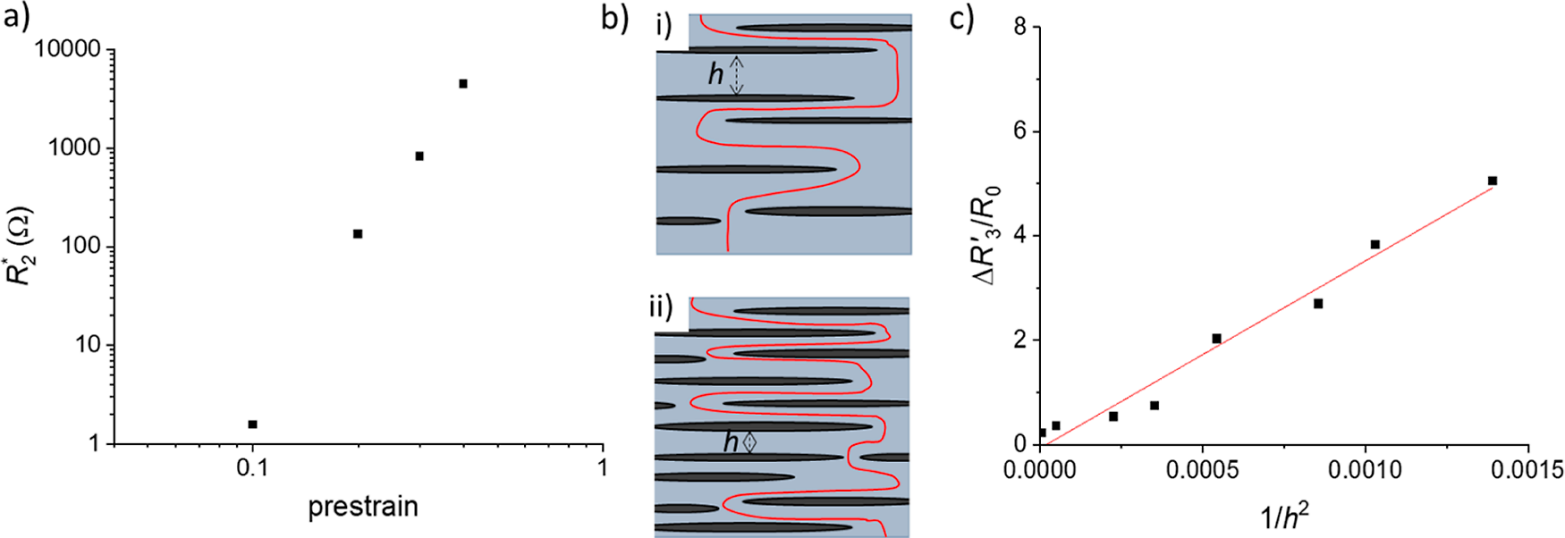
(a) Plot of the mean resistance contributed by an individual channel crack as a function of conditioning prestrain in the 60 s HI-treated rGO/PDMS films that show channel cracking. (b) Schematic illustration showing how a reduction in crack spacing (*h*) of kirigami cracks at (i) low-conditioning strains and (ii) high-conditioning strains leads to an increase in the current path length within the conducting membrane (indicated schematically by a red line). (c) Plot of the change in *R*_3_′ as a function of the square of the inverse of the mean crack spacing in rGO/PDMS films exposed to HI for 30 s and displaying kirigami cracking after strain conditioning.

Figure 7a shows a logarithmic plot of *R*_2_*, the resistance per crack, as a function of the conditioning strain. This suggests that they are related by a power law function with *R*_2_* = *K*ε_0_^*m*^, with *m* = 5.7 and a pre-exponent of *K* = 10^6^. Combining this function for the crack resistance and using the linear relation between the number of cracks and the initial strain (with *n* = *h*/*L*_0_ in eq 5), it is possible to use our data and eqs 4–6 to predict the resistance of the rGO membranes with parallel channel cracks as a function of conditioning prestrain, with
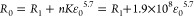
8

The mechanism of electrical conduction across a crack in a conducting film has been considered by others and reviewed recently.^10^ The mechanisms proposed all rest on the assumption that after the initial conditioning strain, the cracks will close through elastic relaxation when the strain is removed. Thus, the open cracks are replaced by two crack surfaces in contact. Because of the details in the film and substrate microstructure, the crack surfaces will not be atomically smooth. Hence, the nature of the contacting asperities on each surface will lead to local, isolated regions of surface face-to-face contact, regions with no contact, and regions where the two films overlap and are in electrical contact out of plane. These are all captured in the increase in resistance of the cracked membrane once the initial conditioning strain, ε_0_, is removed. When the cracked membrane is subsequently strained to strains ε < ε_0_, the crack faces will open but no new cracks will be nucleated. The electrical resistance across the crack faces will increase because of a gradual decrease in the electrical contact across the crack faces.^9,19,37,41^ An alternative mechanism has been proposed where the conduction across the crack face is driven by electron tunneling when the crack opening is very small; however, this would lead to a highly nonlinear piezoresistive response. A transition in mechanism, from asperity contact/film overlap to tunneling, has been proposed by Luo et al.^3^ and also by Yang et al. to explain the transition to higher GF sensing at larger strains.^37^ Hence, we propose that in the channel cracking regime, the series resistor model is consistent with our observations and the resistance of the gauge as a function of strain is described by
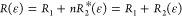
9

Here, the presence of ε in parentheses refers to the previous parameter being strain-dependent, and it is also assumed that no new channel cracks are nucleated during straining. We note that at low conditioning strains, the GF for the channel cracking gauges remains close to 1000, even though the unstrained resistance of the gauges has increased with increasing crack density (Table S3). This supports a common piezoresistive response from the crack opening mechanism in each case. There is, however, considerable variation in the GF as the conditioning strain changes and this may be caused by the stochastic nature of crack nucleation or inconsistencies in the tiled film deposition between batches.

To summarize, when the crack morphology consists of an array of approximately parallel channel cracks in the conducting membrane, the resistance of the membrane under zero load is controlled by the number of cracks normal to the loading direction and the mean electrical resistance of a crack, *R*_2_*. The number of cracks is determined by the elastic interactions between growing cracks with mean crack spacing proportional to the inverse of the initial conditioning strain, ε_0_ (eq 6). The piezoresistive effect is caused by the change in *R*_2_* as the cracked membrane is strained. The large increase in membrane resistance with increasing strain conditioning, ε_0_ is a combination of an increase in the number of cracks and a nonlinear relationship between the resistance of a crack and the maximum crack opening that occurs during strain conditioning. The piezoresistive effect at low strains, ε < ε_0_, is the linear response of the individual resistance of the cracks and this leads to a GF independent of the crack density. The low working strain range of these devices is caused by the mechanism for electrical conductivity across the crack faces having a very small linear range of usable crack opening before an open circuit occurs.

#### Kirigami Cracking

The relationship between membrane resistance and conditioning strain is different for the PDMS/rGO membranes reduced for a shorter period of 30 s, which results in kirigami cracking. Note that in this case the cracks are not continuous, and the mean crack spacing needs to be carefully defined. This is measured by counting the number of cracks that intersect with a line drawn parallel to the loading direction. It represents the mean distance between a crack and its nearest neighbor in that direction and is not the mean separation of all cracks. Comparing the apparent crack spacing as a function of strain with Thouless’s model (eq 5) shows it to be approximately proportional to the inverse of the conditioning strain magnitude (Figure 4b). As with the channel-cracked films, the film resistance increases with increasing ε_0_ but, in contrast, the film resistance remains relatively small (Supporting Information Table S4).

The resistance of the kirigami-cracked membrane is modeled using a series and parallel resistor configuration (eq 3). As with the channel crack model, the resistor *R*_1_ represents the resistance of the uncracked film, and *R*_2_, the resistance of the cracks, but in addition, there is a third resistor, *R*_3_, in parallel with *R*_2_, that represents resistance from the longer current path length caused by the population of discontinuous cracks (Figure 7b). The uncracked film resistance, *R*_1_, is constant while the resistors *R*_2_ and *R*_3_ are assumed to be functions of both the conditioning strain, ε_0_, and the sensed strain, ε. Figure 7a shows that the resistance of the channel cracks increases rapidly with strain, and we expect similar behavior with the individual kirigami cracks. Hence, at large strains, *R*_2_ ≫ *R*_3_ and eq 3 reduces to
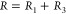
10

**Figure 8:**
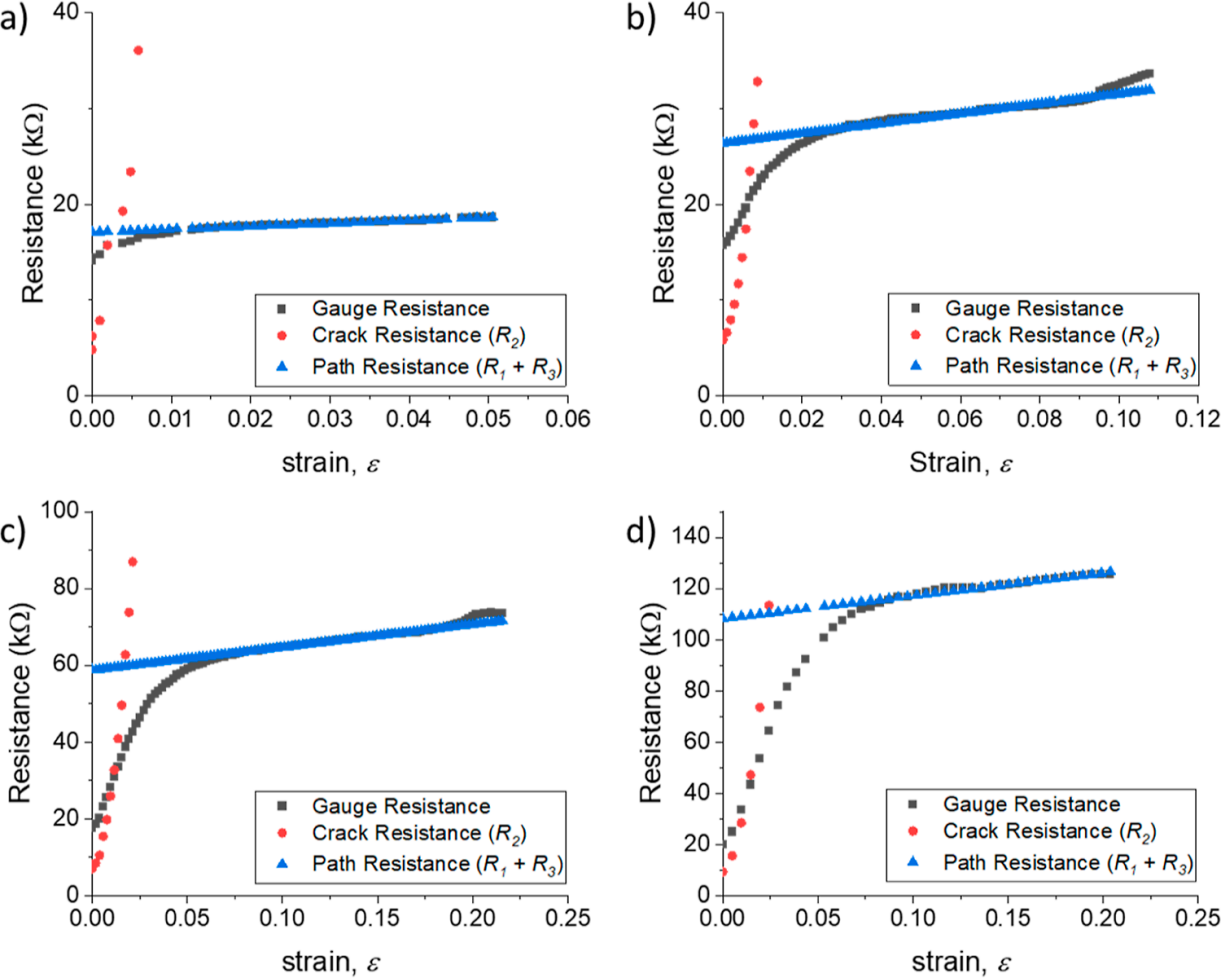
Strain gauge resistance as a function of applied strain separated into the components determined by resistance across a kirigami crack (*R*_2_) and the contribution from the intrinsic gauge resistance and the increased current path length (*R*_1_ + *R*_3_) for gauges with conditioning prestrains (ε_0_) of: (a) 0.05, (b) 0.10, (c) 0.20, and (d) 0.30.

Thus, at large strains, the asymptotic slope of each gauge in Figure 5b must represent the variation of *R*_3_ as a function of strain with
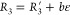
11where *R*_3_′ is the value of *R*_3_ at ε = 0 (the intercept of the asymptotic slope projected to ε = 0), and *b* is the gradient of the asymptotic line or the high strain GF. It also follows from eq 3 that the resistance of the membrane at ε = 0 is given by
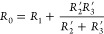
12where *R*_2_′ is the value of *R*_2_ at ε = 0. These can be calculated from the data presented in Figure 5 and the appropriate values of *R*_2_′, *R*_3_′, and *b* are given in the Supporting Information Table S4.

Figure 7b shows a schematic of the kirigami-cracked membrane at large strains, where *R*_2_ ≫ *R*_3_. As the crack density increases, the mean crack separation decreases, as does the mean width of the conducting pathway, which is proportional to *h*, the mean crack spacing. If we assume that the mean crack length remains unchanged, then the path length for conductivity will increase in proportion to 1/*h*. Given that the resistance of the path is proportional to its length and inversely proportional to its width, the increase in resistance, *R*_3_′, will be proportional to 1/*h*^2^, and this is consistent with our measurements (Figure 7c) at conditioning strains, ε_0_ < 0.8. Once strain-conditioned, the membrane will contain a distribution of discontinuous, approximately parallel cracks. At zero strain, the cracks will close by relaxation, and the crack bridging resistance is small. We assume that subsequent straining as a strain gauge, the cracks generated during conditioning open but do not grow any longer and that no new cracks are nucleated if ε < ε_0_. The resistance of the strain gauge increases from two contributions: a linear increase in *R*_3_ as described by the measured gradient *b* and an increase in the crack bridging resistance that occurs following the kirigami opening of the cracks during straining. This change in resistance is expected to increase rapidly with strain following the behavior observed with the channel crack devices. From inspection of Figure 5b and the data presented in Supporting Information Table S4, the GF at large strains is very similar for all conditioning strains <0.4. We interpret this as indicating that the change in *R*_3_ with strain is independent of the conditioning strain, whereas the initial value after conditioning, *R*_3_′, is strongly dependent on the conditioning strain. We propose that the change in *R*_3_ with strain is caused by a gradual decrease in the edge-to-edge contact of the rGO flakes within the conducting film.

The performance of the strain gauge can be interpreted using a modified form of eq 12
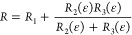
13where *R*_2_(ε) and *R*_3_(ε) are the change in crack resistance and the change in resistance of the increased path length, as a function of the applied strain, respectively. Figure 8 uses eq 13 to show the response of the gauge and its separation into contributions from *R*_2_(ε) and *R*_3_(ε). This demonstrates that the characteristic form of the strain response curve is shown to be a consequence of the low initial resistance of the closed kirigami cracks, *R*_2_′, in parallel with resistance caused by the longer path length after kirigami cracking *R*_3_′. As strain increases, *R*_2_(ε), grows much more rapidly than *R*_3_(ε), and thus, at high strains, the strain response asymptotes to the increase in resistance determined by the *R*_3_(ε). We define the asymptotic strain, ε_3_, when the measured gauge resistance reaches 99% of the asymptotic value. This is shown in Table S3 along with the fraction of the conditioning strain, ε_3_/ε_0_, at which this is observed. At all conditioning strains <0.4, the transition to the asymptotic behavior with a constant GF occurs at about ε_3_/ε_0_ = 0.3, with a constant GF of approximately 3 above this strain.

Thus, as with the channel cracking membranes, the initial strain response is controlled by the very steep increase in the crack electrical resistance as the cracks open during straining. The main difference between the channel cracking and kirigami cracking gauges is that the crack opening resistance is in series with the resistance contribution from the intact membrane when channel cracks form and is in parallel with the membrane resistance when kirigami cracking occurs. The parallel resistance configuration of the kirigami-cracked films allows a useful piezoresistive effect to continue to large strains, while the series configuration of the channel-cracked films swiftly rises to an open circuit value, beyond which it is ineffective. At large strains with the kirigami cracking gauges, it is proposed that the increase in resistance comes from the gradual reduction in edge-to-edge contact within the densely packed tiled 2D monolayer formed during the deposition process. Although this results in a relatively low value of GF, it is comparable to the GF seen with conventional thin film metal strain gauges, however, with a mechanism that differs from both the reduction in current carrying area and the increase in path length that occurs with conventional strain gauges. The piezoresistive response at large strains is possible because of the kirigami nature of the multiple cracks that form, reducing the likelihood of large channel cracks forming and consequent creation of open circuit conditions. In all cases when the kirigami gauges stopped working at high strains, this was accompanied by the nucleation of channel cracks. Hence, a better understanding of the conditions that promote kirigami cracking rather than channel cracking is needed to design crack-based gauges that can operate reliably over large strain ranges. The poor performance of the kirigami gauges at larger (>0.4) conditioning strains may also be caused by the introduction of channel cracks or possibly by the wrinkling that was reported earlier when ε_0_ > 0.2 (Figure S5), which could lead to local decohesion of the tiled films.

## Conclusions

We have demonstrated that it is possible to develop a transparent crack-based strain gauge with a high GF from a monolayer of GO flakes, tiled with good edge-to-edge contact and minimal surface overlap, deposited on a thin PDMS elastomeric film. In order to achieve appropriate electrical resistance, the GO film must be reduced in the presence of HI vapor to form a rGO/PDMS membrane. After reduction, the rGO films retain their transparency, allowing 88% of incident light to pass through. The films also show a high TCE figure of merit (ratio of electrical-to-optical conductivity) and, as shown in Figure 3b, show performance comparable with the best literature values despite being processed at much lower temperatures.

The membranes need to be conditioned by straining elastically by a value of ε_0_ before use as strain gauges. This conditioning process is required to generate a population of parallel cracks, needed to introduce a piezoresistive response. The mean spacing of the cracks decreases in inverse proportion to the applied conditioning strain, in accordance with simple mechanical models of periodic cracking in brittle thin films. However, crack morphology after conditioning, and consequent strain gauge performance, is strongly dependent on the reducing treatment used in gauge production. After 30 s reduction by HI, the cracks that form during initial straining arrest before spanning the specimen. Subsequent straining after relaxation to strains ε < ε_0_ leads to a kirigami opening of the cracks without further extension. However, if the reduction is allowed to proceed for 60 s, then the conditioning strain leads to the formation of channel cracks that propagate across the entire width of the specimen. Both of these cracking morphologies form in rGO films that have high optical transmittance and can be used as transparent strain gauges. The channel cracking configuration results in a very high sensitivity strain gauge with GF > 10,000 but with operating maximum strain, ε < 0.01. The kirigami cracking gauges show a nonlinear but repeatable response up to the initial conditioning strain level when ε_0_ is ≤0.4. The GF reduces with increasing strain until reaching a value of GF ≈ 3, when the applied strain is about 50% of the initial conditioning prestrain. With ε_0_ > 0.3, there is still a useful operating level but at a reduced strain range. The limiting operating strain for the kirigami devices is either the conditioning strain or the strain at which unwanted channel cracks nucleate, and this nucleation is more likely when ε_0_ > 0.3.

Human motion sensing is achievable by these sensors due to their low value of Young’s modulus, *E* ≈ 2 MPa,^42^ being comparable with that of skin (*E* = 0.05–20 MPa).^43,44^ The strain sensors reported here are ideal candidates for human motion sensing applications in, e.g., e-skins, medical diagnostics, and human-machine interfacing. The PDMS membranes are biocompatible and body conformable, ensuring maximum user comfort, and the high transparency of the devices enables invisible strain sensing. Moreover, the GF and sensing range of the devices can be tuned over almost 2 orders of magnitude to suit applications where maximum sensitivity is required, such as structural health monitoring. However, further optimization of the gauges is required to ensure uniform and repeatable performance.

## Supporting Information Available

Schematic and photographs highlighting the importance of sufficiently high surface energy on GO monolayer deposition; experimental setup for the deposition of GO monolayers; method and table of results from a statistical analysis of rGO film coverage by SEM image contrast thresholding; SEM images of channel and kirigami crack morphologies; wrinkling in rGO/silica-like layer due to Poisson compression; piezoresistive stability of kirigami strain sensor over 6000 strain-release cycles of 5%; table of electrical and optical conductivity ratio for rGO TCEs found in the literature; table of electrical resistance and crack spacing of PDMS/rGO membranes reduced for 60 s; table of electrical resistance and crack spacing of PDMS/rGO membranes reduced for 30 s; and table of literature examples of film-based strain gauges and their performance (PDF)
