# Supplementary material for: Graphene-Based Transparent Flexible Strain Gauges with Tunable Sensitivity and Strain Range
Source: ACS Appl Nano Mater. 2023 Nov 22;6(23):21763–74. doi: 10.1021/acsanm.3c03967 (PMC10714313; doi:10.1021/acsanm.3c03967)
Supplement: Supplementary file 1 — an3c03967_si_001.pdf [file an3c03967_si_001.pdf]

Supporting Information

Graphene Based Transparent Flexible Strain Gauges with Tuneable Sensitivity and Strain Range

Joseph Neilson, [1, 2] *Pietro Cataldi,* [1, 3] *and Brian Derby* [1]*

1. Department of Materials, University of Manchester, Oxford Road, Manchester M13 9PL, UK.
2. Department of Physics, Trinity College Dublin, Dublin, Ireland.
3. Smart Materials, Istituto Italiano di Tecnologia, Via Morego 30, Genova, 16163, Italy.

*Corresponding Author

E-mail: brian.derby@manchester.ac.uk

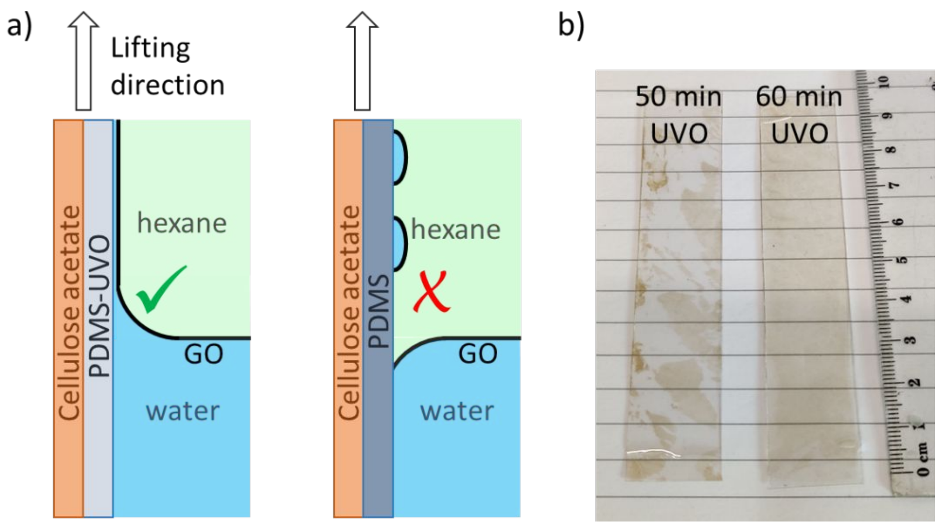

Figure S1 a) Schematic representation of the continuous deposition of GO via liquid-liquid interface assembly. *Left panel*: the deposition of a continuous GO film relies on a small 3-phase contact angle between the substrate, water, and hexane. *Right panel*: a large 3-phase contact angle will result in beading of the water layer. b) *Left panel*: 50 minute UV-ozone treated PDMS/GO film deposited with a large contact angle showing patchy GO film reduced to rGO. *Right panel*: 60 minute UV-ozone treated PDMS/GO film deposited with a small contact angle showing a uniform GO film reduced to rGO.

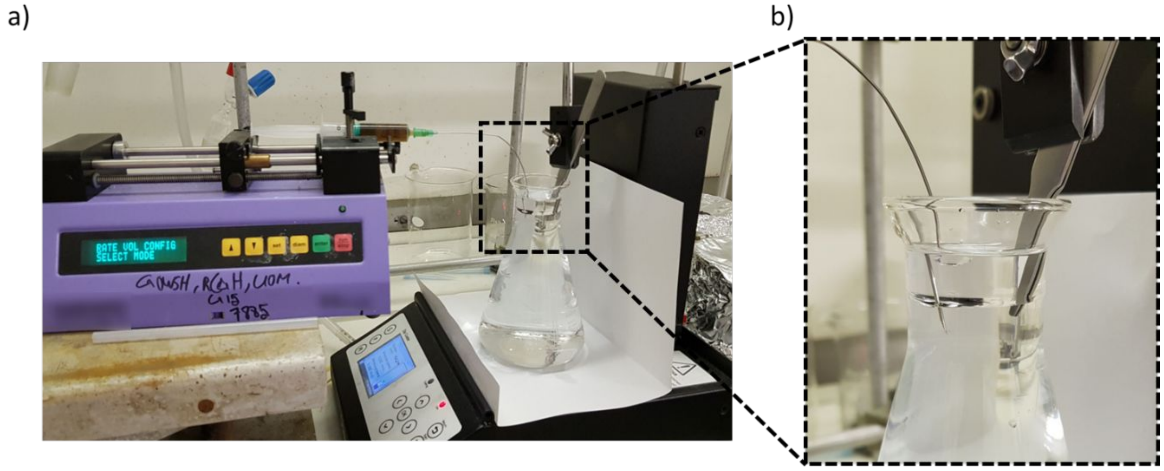

Figure S2 a) Experimental setup for the continuous production of liquid-liquid assembled monolayers of GO. b) GO film on PDMS. The black box indicates the projected area of the liquid-liquid interface (7.1 cm^2) used in the deposition of the GO film of area 25 cm^2.

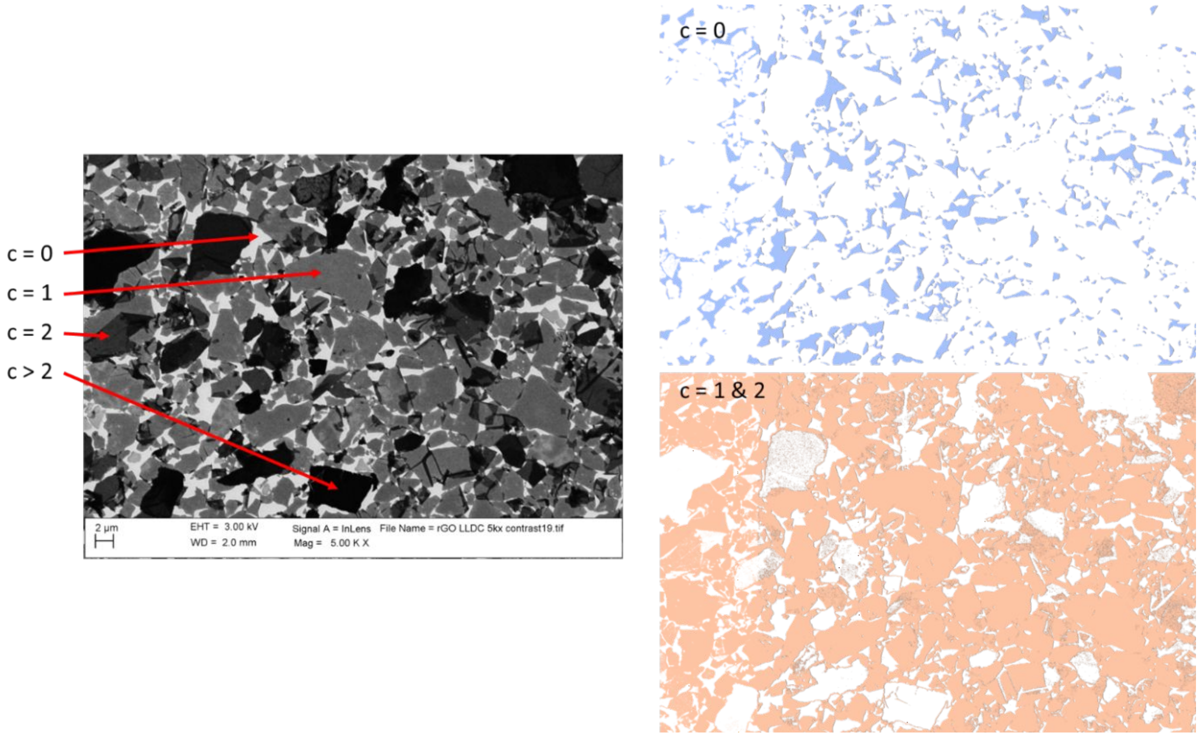

Figure S3 SEM image of an rGO film deposited by liquid-interface assembly (left panel). The image can be separated into zero coverage (c = 0, top right panel), mono and bi-layer coverage (c = 1 & 2, bottom right panel) and stacked coverage (c > 2) by thresholding in the ImageJ software. [1]

The thresholding procedure as described in Figure S3 was used in the statistical analysis of 3 x SEM images of rGO films, demonstrating the predominantly mono- and bi-layer coverage in the films.

Table S1 Statistical analysis of the contrast in SEM images, demonstrating the predominantly monolayer/bilayer coverage of our rGO films.

**Image Number**	**Zero Coverage, c = 0 (%)**	**Mono/Bi-Layer Coverage, c = 1 & 2 (%)**	**Stacked, c > 2 (%)**
1	5.2	62.1	32.7
2	8.1	64.8	27.1
3	11.8	67.1	21.1
**Average**	8.4 ± 2.7	64.7 ± 2.0	26.9 ± 4.7

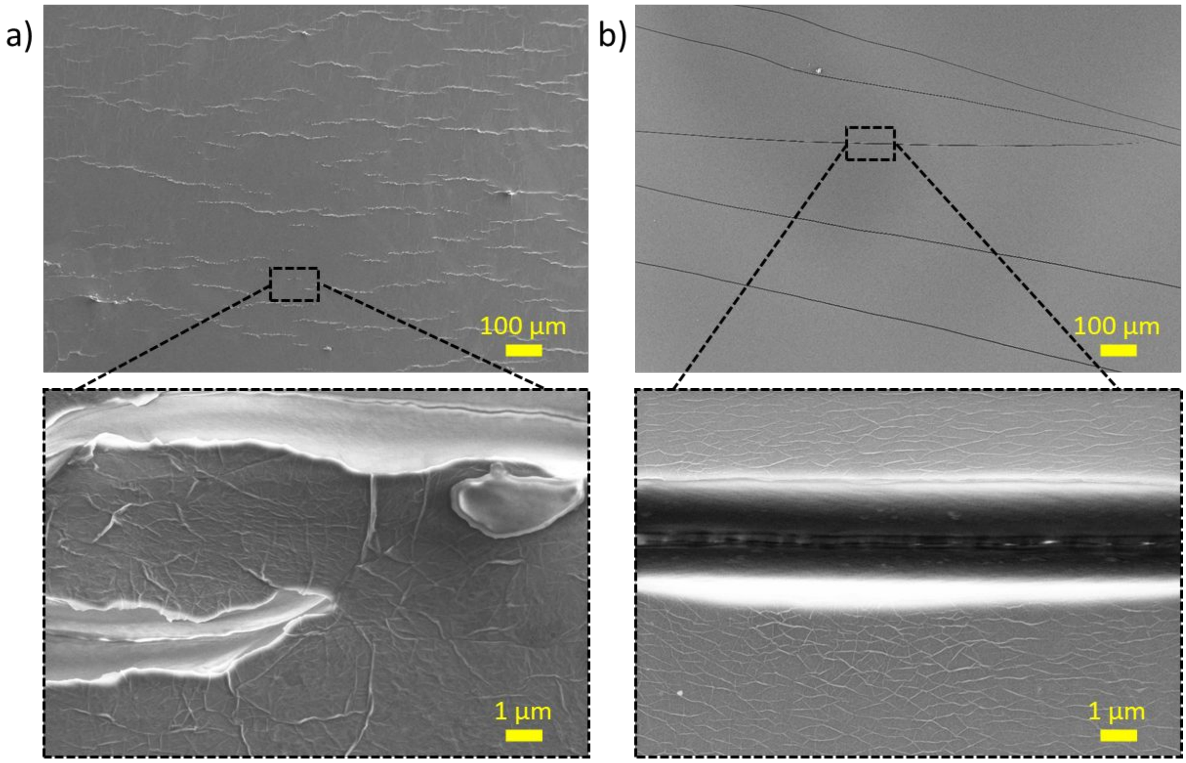

Figure S4: SEM images of crack morphologies a) kirigami and b) channel cracks. High magnification images are included in the bottom panels, indicated by the dashed black boxes. Each gauge was prestrained at ε_0 = 0.2. The channel gauge is mounted at ε = 0.01 and the kirigami gauge is mounted at strain ε = 0.1

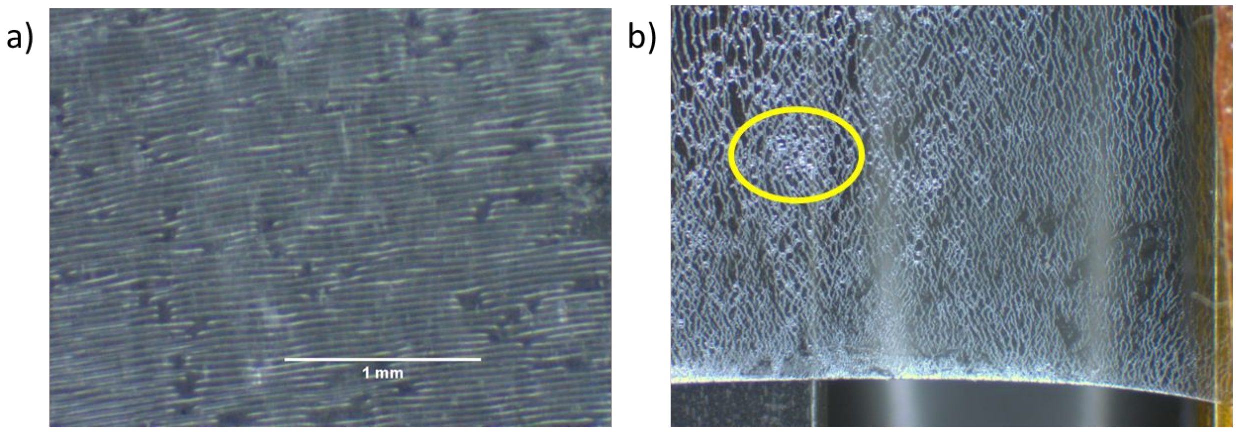

Figure S5 a) Wrinkling in silica-like layer under compressive strain of 0.2. This was obtained by straining a PDMS membrane to $\varepsilon = 0.2$, performing 1 hour UV-ozone plasma treatment on and releasing the strain. b) Wrinkling/delamination of rGO film parallel to strain direction under 30 % strain

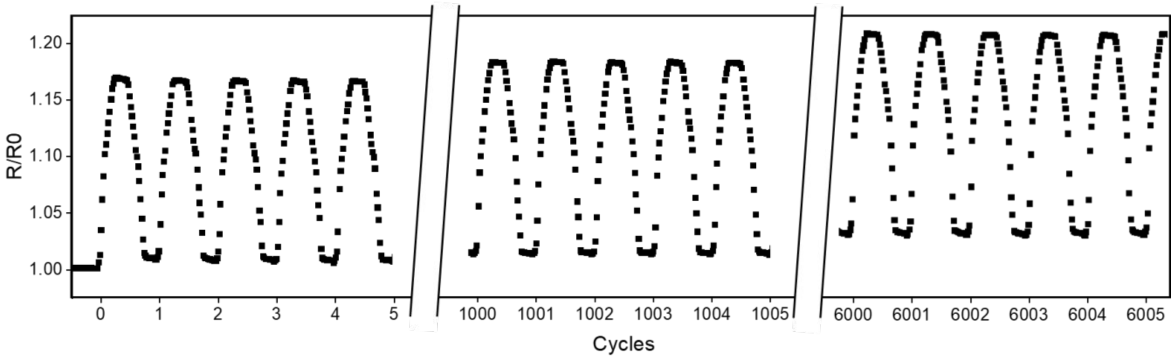

Figure S6 piezoresistive response of kirigami strain sensor over 6000 strain-release cycles of 5 %.

Table S2 Optical transmission, sheet resistance, and corresponding conductivity ratio, $\frac{\sigma_{DC}}{\sigma_{op}}$ for TCE rGO films previously reported in the literature**.**

GO Production method	rGO Formation method (Reduction Process)	Film Deposition method	T at 550 nm %	R_s (Ω/□)	$\frac{\sigma_{DC}}{\sigma_{op}}$	Ref.
Electrochemical	HI Vapour 70 °C 5 mins	Liquid/liquid continuous	**88**	**850**	**3.36**	**This work**
Modified Hummers	Thermal 1100 °C	Spin	80	1000	1.59	2
Modified Hummers	Hydrazine vapour 400 °C	Spin	80	10000	0.159	2
Modified Hummers	Chemical: Nascent H_2 released by etching of Al with HCl	Spin	84.5	20460	0.104	3
Hummers	Thermal 1100 °C	Spin	82	800	2.25	4
Hummers	Thermal 1100 °C under Vacuum	Spin	80	5000	0.319	5
Hummers	Thermal 1100 °C under Ar and acetylene	Spin	70	1750	0.552	6
Modified Brodie	NaBH4 solution (150mM)	Spray	81	4400	0.386	7
Modified Brodie	NaBH4 and AuCl doped	Spray	81	2100	0.808	7
Modified Hummers	Dispersion containing GO reduced with H2 gas at 50 bar	Spray	80	7500	0.213	8
Modified Brodie	Roll to roll, SnCl2/EtOH sprayed and 60 °C	Spray	82.9	800	2.39	9
Ethylbenzoic acid functionalisation of graphite	Thermal 600° C in Ar	Drop cast	90	3110	1.12	10
Hummers	Thermal 1100 °C under H2 Ar	Dip coating	70	1800	0.536	11
Hummers	Thermal 1100 °C under Ar	Dip coating	70	8000	0.121	12

**Table S2 continued**						
**GO Production method**	**rGO Formation method (Reduction Process)**	**Film Deposition method**	**T at 550 nm %**	**R_s (Ω/□)**	$\frac{\sigma_{DC}}{\sigma_{op}}$	**Ref.**
Modified Hummers	HI Solution (55%)<br>100 °C	Liquid/air (induced by heating)	78	840	1.69	13
Hummers	Chemical Hydrazine monohydrate 80 °C	Liquid/air (induced by reduction)	87	11300	0.231	14
Modified Hummers	Immersion in 55% HI 100 °C	Liquid-air (induced by heating)	85	1600	1.39	15
Modified Hummers with thermal expansion at 1050 °C	Thermally 1100 °C Chemically doped with HNO3 and SOCl2	Langmuir-Blodgett	90	459	7.59	16
Modified Hummers	Thermal (1100 C)<br>Under Ar	Langmuir-Blodgett	86	605	3.98	17
Modified Hummers	Hydrazine 70 °C	Pentane-water assembly	72	8300	0.127	18
Modified Hummers	Hydrazine (5 %) 95 °C	Toluene-water (induced by reduction)	70	1800	0.536	19
Electrochemical intercalation and oxidation of graphite foil	HI and acetic acid vapour.	Inkjet printing	92.8	14200	0.349	20

Table S3: Electrical resistance and crack spacing of PDMS/rGO membranes reduced for 60 s (channel cracking), measured after applying a conditioning strain, ε_0, and reducing the strain to zero. Specimen gauge length, L_0 = 12.6 mm.

Conditioning Strain (ε_o)	Mean Crack Spacing (h) μm	Crack No. (n)	Membrane Resistance (R) kΩ	Resistance Increase kΩ	Resistance per Crack (R^*_2) Ω	GF Low Strain	GF High Strain	OC Strain	Transition Strain
0			9.3			930 ± 280	n/a	0.0024	
0.1	396	32	9.3	0.05	1.6	980 ± 120	n/a	0.0048	
0.2	264	48	15.6	6.35	133	1020 ± 140	16600	0.0064	0.005
0.3	205	61	60.0	5.75	826	1160 ± 250	18000	0.0056	0.004
0.4	196	64	298.0	289	4491	770 ± 240	n/a	0.0024	

Table S4: Electrical resistance and crack spacing of PDMS/rGO membranes reduced for 30 s (kirigami), measured after different levels of conditioning strain. Specimen gauge length, L_0 = 10.3 mm.

Conditioning Strain (ε_o)	Mean Crack Spacing (h) μm	Membrane Resistance (R_0) kΩ	Resistance Increase kΩ	GF Low Strain	GF High Strain	Transition Strain (ε_3)	$\frac{\varepsilon_3}{\varepsilon_0}$	R'_2 kΩ	R'_3 kΩ
0	-	11.5	-	3.7	-				
0.05	419	14.1	2.6	23.4	2.2 ± 0.05	0.015	0.33	4.8	5.6
0.1	156	15.7	4.2	40.3	3.3 ± 0.2	0.03	0.30	5.8	14.8
0.2	80	17.6	6.1	65.0	3.4 ± 0.03	0.07	0.35	7.0	47.3
0.3	69	20.0	8.5	86.8	4.5 ± 0.2	0.09	0.30	9.4	96.9
0.4	60	34.8	23.3	97.2	2.1 ± 0.3	0.10	0.25	26.1	218.4
0.5	51	42.5	31.0	113.3	4.0 ± 2.2	0.15	0.3	33.4	432.5
0.6	50	55.6	44.1	148.8	15.7 ± 5.1	-		46.4	876.7
0.8	48	69.6	58.1	-	-				
1.0	50	156.9	145.4	-	-				

Table S5: Literature examples of film based strain gauges showing, strain range, gauge factor and optical transparency, for comparison with our results.

**Material type**	**strain range**	**GF (max)**	**Transparency (%)**	**Sensing mechanism**	**ref**
**2D**	**0.006**	**16000**	**88**	**Crack based (transverse)**	**This Work**
**2D**	0.2	**285**	**88**	**Crack based (kirigami)**	**This Work**
2D	0.06	1000	NA	Crack based	21
2D	0.071	14	80	Crack/defect based	22
2D	NA	4.46	no	Crack based	23
2D	0.5	630	NA	Changing contact resistance	24
2D	0.004	300	NA	Charge tunnelling	25
2D	0.06	66.6	No	Crack based	26
2D	0.3	0.55	NA	Structural deformation of graphene	27
2D	NA	15	no	Flake-flake overlap modulation	28
2D	1	10	NA	Percolation modulation	29
2D	0.1	9.5	no	Crack based	30
2D	80	20	no	Percolation modulation	31
2D	800	35	no	Percolation modulation	32
2D	500	NA	no	Dynamic percolation modulation	33
2D	10	2	NA	Crack based	34
2D	2	137	no	Percolation modulation	35

**Table S5 continued**					
**Material type**	**strain range**	**GF (max)**	**Transparency (%)**	**Sensing mechanism**	**Ref**
2D	0.283	3.82	No	Percolation modulation	36
2D	26	1054	No	Crack based	37
2D	550	6583	No	Percolation modulation	38
2D	20	42.2	89.1	Crack based	39
2D	25	35	No	Crack based	40
2D	0.5	139	NA	Percolation modulation	41
1D	1	4000	No	Crack based	42
1D	30	84.6	86.3	Crack based	43
1D	100	30	90	crack based	44
1D	30	200	92	Percolation modulation	45
1D	150	846	88	Percolation modulation	46
1D	100	62.3	62	Percolation modulation	47
Metal	2	4000	89	Crack based	48
Metal	2	2000	No	Crack based	49
Metal	2	5000	No	Crack based	50
Metal	2	1600	No	Crack based	51
Metal	7	10000	No	Crack based	52
Metal	2	10000	No	Crack based	53

Bibliography

(1) Schindelin, J.; Arganda-Carreras, I.; Frise, E.; Kaynig, V.; Longair, M.; Pietzsch, T.; Preibisch, S.; Rueden, C.; Saalfeld, S.; Schmid, B.; et al. Fiji: An open-source platform for biological-image analysis. *Nature Methods*, **2012**, 9, 676-682.

(2) Becerril, H. A.; Mao, J.; Liu, Z.; Stoltenberg, R. M.; Bao, Z.; Chen, Y. Evaluation of solution-processed reduced graphene oxide films as transparent conductors. *ACS Nano* **2008**, *2*, 463-470.

(3) Domingues, S. H.; Kholmanov, I. N.; Kim, T.; Kim, J.; Tan, C.; Chou, H.; Alieva, Z. A.; Piner, R.; Zarbin, A. J. G.; Ruoff, R. S. Reduction of graphene oxide films on Al foil for hybrid transparent conductive film applications. *Carbon* **2013**, *63*, 454-459.

(4) Wu, J.; Agrawal, M.; Becerril, H. A.; Bao, Z.; Liu, Z.; Chen, Y.; Peumans, P. Organic light-emitting diodes on solution-processed graphene transparent electrodes. *ACS Nano* **2010**, *4*, 43-48.

(5) Wu, J.; Becerril, H. A.; Bao, Z.; Liu, Z.; Chen, Y.; Peumans, P. Organic solar cells with solution-processed graphene transparent electrodes. *Appl. Phys. Lett.* **2008**, *92*.

(6) Liang, Y.; Frisch, J.; Zhi, L.; Norouzi-Arasi, H.; Feng, X.; Rabe, J. P.; Koch, N.; Müllen, K. Transparent, highly conductive graphene electrodes from acetylene-assisted thermolysis of graphite oxide sheets and nanographene molecules. *Nanotechnology* **2009**, *20*.

(7) Shin, H. J.; Kim, K. K.; Benayad, A.; Yoon, S. M.; Park, H. K.; Jung, I. S.; Jin, M. H.; Jeong, H. K.; Kim, J. M.; Choi, J. Y.; et al. Efficient reduction of graphite oxide by sodium borohydride and its effect on electrical conductance. *Adv. Funct. Mater.* **2009**, *19*, 1987-1992.

(8) Liang, M.; Wang, J.; Luo, B.; Qiu, T.; Zhi, L. High-efficiency and room-temperature reduction of graphene oxide: A facile green approach towards flexible graphene films. *Small* **2012**, *8*, 1180-1184.

(9) Ning, J.; Hao, L.; Jin, M.; Qiu, X.; Shen, Y.; Liang, J.; Zhang, X.; Wang, B.; Li, X.; Zhi, L. A Facile Reduction Method for Roll-to-Roll Production of High Performance Graphene-Based Transparent Conductive Films. *Advanced Materials* **2017**, *29*.

(10) Bae, S. Y.; Jeon, I. Y.; Yang, J.; Park, N.; Shin, H. S.; Park, S.; Ruoff, R. S.; Dai, L.; Baek, J. B. Large-area graphene films by simple solution casting of edge-selectively functionalized graphite. *ACS Nano* **2011**, *5*, 4974-4980.

(11) Wang, X.; Zhi, L.; Müllen, K. Transparent, conductive graphene electrodes for dye-sensitized solar cells. *Nano Letters* **2008**, *8*, 323-327.

(12) Zhao, L.; Zhao, L.; Xu, Y.; Qiu, T.; Zhi, L.; Shi, G. Polyaniline electrochromic devices with transparent graphene electrodes. *Electrochimica Acta* **2009**, *55*, 491-497.

(13) Zhao, J.; Pei, S.; Ren, W.; Gao, L.; Cheng, H. M. Efficient preparation of large-area graphene oxide sheets for transparent conductive films. *ACS Nano* **2010**, *4*, 5245-5252.

(14) Zhu, Y.; Cai, W.; Piner, R. D.; Velamakanni, A.; Ruoff, R. S. Transparent self-assembled films of reduced graphene oxide platelets. *Appl. Phys. Lett.* **2009**, *95*.

(15) Pei, S.; Zhao, J.; Du, J.; Ren, W.; Cheng, H. M. Direct reduction of graphene oxide films into highly conductive and flexible graphene films by hydrohalic acids. *Carbon* **2010**, *48*, 4466-4474.

(16) Zheng, Q.; Ip, W. H.; Lin, X.; Yousefi, N.; Yeung, K. K.; Li, Z.; Kim, J. K. Transparent conductive films consisting of ultralarge graphene sheets produced by Langmuir-Blodgett assembly. In *ACS Nano*, **2011**, 5, 6039-6051.

(17) Zheng, Q.-b.; Shi, L.-f.; Yang, J.-h. Langmuir-Blodgett assembly of ultra-large graphene oxide films for transparent electrodes. *Trans. Nonferrous Metals Soc. China* **2012**, *22*, 2504-2511.

(18) Chen, F.; Liu, S.; Shen, J.; Wei, L.; Liu, A.; Chan-Park, M. B.; Chen, Y. Ethanol-assisted graphene oxide-based thin film formation at pentane-water interface. *Langmuir* **2011**, *27*, 9174-9181.

(19) Gan, S.; Zhong, L.; Wu, T.; Han, D.; Zhang, J.; Ulstrup, J.; Chi, Q.; Niu, L. Spontaneous and fast growth of large-area graphene nanofilms facilitated by oil/water interfaces. *Adv. Mater.* **2012**, *24*, 3958-3964.

(20) Cao, J.; He, P.; Mohammed, M. A.; Zhao, X.; Young, R. J.; Derby, B.; Kinloch, I. A.; Dryfe, R. A. W. Two-Step Electrochemical Intercalation and Oxidation of Graphite for the Mass Production of Graphene Oxide. *J. Amer. Chem. Soc.* **2017**, *139*, 17446-17456.

(21) Li, X.; Zhang, R.; Yu, W.; Wang, K.; Wei, J.; Wu, D.; Cao, A.; Li, Z.; Cheng, Y.; Zheng, Q.; et al. Stretchable and highly sensitive graphene-on-polymer strain sensors. *Sci. Rep.* **2012**, *2*.

(22) Bae, S. H.; Lee, Y.; Sharma, B. K.; Lee, H. J.; Kim, J. H.; Ahn, J. H. Graphene-based transparent strain sensor. *Carbon* **2013**, *51*, 236-242.

(23) Yuan, W.; Zhou, Q.; Li, Y.; Shi, G. Small and light strain sensors based on graphene coated human hairs. *Nanoscale* **2015**, *7*, 16361-16365.

(24) Tang, Y.; Zhao, Z.; Hu, H.; Liu, Y.; Wang, X.; Zhou, S.; Qiu, J. Highly Stretchable and Ultrasensitive Strain Sensor Based on Reduced Graphene Oxide Microtubes-Elastomer Composite. *ACS Appl. Mater. Interf.* **2015**, *7*, 27432-27439.

(25) Zhao, J.; He, C.; Yang, R.; Shi, Z.; Cheng, M.; Yang, W.; Xie, G.; Wang, D.; Shi, D.; Zhang, G. Ultra-sensitive strain sensors based on piezoresistive nanographene films. *Appl. Phys. Lett.* **2012**, *101*.

(26) Saha, B.; Baek, S.; Lee, J. Highly Sensitive Bendable and Foldable Paper Sensors Based on Reduced Graphene Oxide. *ACS Appl. Mater. Interf.* **2017**, *9*, 4658-4666.

(27) Wang, Y.; Yang, R.; Shi, Z.; Zhang, L.; Shi, D.; Wang, E.; Zhang, G. Super-Elastic Graphene Ripples for Flexible Strain Sensors. *ACS Nano* **2011**, *5*, 3645-3650.

(28) Hempel, M.; Nezich, D.; Kong, J.; Hofmann, M. A Novel Class of Strain Gauges Based on Layered Percolative Films of 2D Materials. *Nano Lett.* **2012**, *12*, 5714-5718.

(29) Park, J. J.; Hyun, W. J.; Mun, S. C.; Park, Y. T.; Park, O. O. Highly stretchable and wearable graphene strain sensors with controllable sensitivity for human motion monitoring. *ACS Appl. Mater. Interf.* **2015**, *7*, 6317-6324.

(30) Tian, H.; Shu, Y.; Cui, Y.-L.; Mi, W.-T.; Yang, Y.; Xie, D.; Ren, T.-L. Scalable fabrication of high-performance and flexible graphene strain sensors. *Nanoscale* **2014**, *6*, 699-705.

(31) O'Mara, M. A.; Ogilvie, S. P.; Large, M. J.; Amorim Graf, A.; Sehnal, A. C.; Lynch, P. J.; Salvage, J. P.; Jurewicz, I.; King, A. A. K.; Dalton, A. B. Ultrasensitive Strain Gauges Enabled by Graphene-Stabilized Silicone Emulsions. *Adv. Funct. Mater.* **2020**, *30*, 2002433.

(32) Boland, C. S.; Khan, U.; Backes, C.; O'Neill, A.; McCauley, J.; Duane, S.; Shanker, R.; Liu, Y.; Jurewicz, I.; Dalton, A. B.; et al. Sensitive, high-strain, high-rate bodily motion sensors based on graphene-rubber composites. *ACS Nano* **2014**, *8*, 8819-8830.

(33) Boland, C. S.; Khan, U.; Ryan, G.; Barwich, S.; Charifou, R.; Harvey, A.; Backes, C.; Li, Z.; Ferreira, M. S.; Möbius, M. E.; et al. Sensitive electromechanical sensors using viscoelastic graphene-polymer nanocomposites. *Science* **2016**, *354*, 1257-1260.

(34) Yang, T.; Jiang, X.; Zhong, Y.; Zhao, X.; Lin, S.; Li, J.; Li, X.; Xu, J.; Li, Z.; Zhu, H. A wearable and highly sensitive graphene strain sensor for precise home-based pulse wave monitoring. *ACS Sensors* **2017**, *2*, 967-974.

(35) Coskun, M. B.; Akbari, A.; Lai, D. T. H.; Neild, A.; Majumder, M.; Alan, T. Ultrasensitive Strain Sensor Produced by Direct Patterning of Liquid Crystals of Graphene Oxide on a Flexible Substrate. *ACS Appl. Mater. Interf.* **2016**, *8*, 22501-22505.

(36) Qi, X.; Li, X.; Jo, H.; Sideeq Bhat, K.; Kim, S.; An, J.; Kang, J.-W.; Lim, S. Mulberry paper-based graphene strain sensor for wearable electronics with high mechanical strength. *Sens. Actuators A Phys.* **2020**, *301*, 111697.

(37) Yang, Y.-F.; Tao, L.-Q.; Pang, Y.; Tian, H.; Ju, Z.-Y.; Wu, X.-M.; Yang, Y.; Ren, T.-L. An ultrasensitive strain sensor with a wide strain range based on graphene armour scales. *Nanoscale* **2018**, *10*, 11524-11530.

(38) Jia, Y.; Yue, X.; Wang, Y.; Yan, C.; Zheng, G.; Dai, K.; Liu, C.; Shen, C. Multifunctional stretchable strain sensor based on polydopamine/ reduced graphene oxide/ electrospun thermoplastic polyurethane fibrous mats for human motion detection and environment monitoring. *Compos. B: Eng.* **2020**, *183*, 107696.

(39) Chun, S.; Choi, Y.; Park, W. All-graphene strain sensor on soft substrate. *Carbon* **2017**, *116*, 753-759.

(40) Liu, Y.; Zhang, D.; Wang, K.; Liu, Y.; Shang, Y. A novel strain sensor based on graphene composite films with layered structure. *Compos. A Appl. Sci. Manuf.* **2016**, *80*, 95-103.

(41) Gao, F.; Qiu, Y.; Wei, S.; Yang, H.; Zhang, J.; Hu, P. Graphene nanoparticle strain sensors with modulated sensitivity through tunneling types transition. *Nanotechnology* **2019**, *30*, 425501.
(42) Chen, S.; Wei, Y.; Wei, S.; Lin, Y.; Liu, L. Ultrasensitive Cracking-Assisted Strain Sensors Based on Silver Nanowires/Graphene Hybrid Particles. *ACS Appl. Mater. Interf.* **2016**, *8*, 25563-25570.
(43) Yin, F.; Lu, H.; Pan, H.; Ji, H.; Pei, S.; Liu, H.; Huang, J.; Gu, J.; Li, M.; Wei, J. Highly Sensitive and Transparent Strain Sensors with an Ordered Array Structure of AgNWs for Wearable Motion and Health Monitoring. *Sci. Rep.* **2019**, *9*, 2403.
(44) Lee, C.-J.; Park, K. H.; Han, C. J.; Oh, M. S.; You, B.; Kim, Y.-S.; Kim, J.-W. Crack-induced Ag nanowire networks for transparent, stretchable, and highly sensitive strain sensors. *Sci. Rep.* **2017**, *7*.
(45) Lee, J.; Lim, M.; Yoon, J.; Kim, M. S.; Choi, B.; Kim, D. M.; Kim, D. H.; Park, I.; Choi, S.-J. Transparent, Flexible Strain Sensor Based on a Solution-Processed Carbon Nanotube Network. *ACS Appl. Mater. Interf.* **2017**, *9*, 26279-26285.
(46) Wang, Z.; Zhang, L.; Liu, J.; Li, C. Highly Stretchable, Sensitive, and Transparent Strain Sensors with a Controllable In-Plane Mesh Structure. *ACS Appl. Mater. Interf.* **2019**, *11*, 5316-5324.
(47) Roh, E.; Hwang, B. U.; Kim, D.; Kim, B. Y.; Lee, N. E. Stretchable, Transparent, Ultrasensitive, and Patchable Strain Sensor for Human-Machine Interfaces Comprising a Nanohybrid of Carbon Nanotubes and Conductive Elastomers. *ACS Nano* **2015**, *9*, 6252-6261.
(48) Lee, T.; Choi, Y. W.; Lee, G.; Pikhitsa, P. V.; Kang, D.; Kim, S. M.; Choi, M. Transparent ITO mechanical crack-based pressure and strain sensor. *J. Mater. Chem. C* **2016**, *4*, 9947-9953.
(49) Kang, D.; Pikhitsa, P. V.; Choi, Y. W.; Lee, C.; Shin, S. S.; Piao, L.; Park, B.; Suh, K. Y.; Kim, T. I.; Choi, M. Ultrasensitive mechanical crack-based sensor inspired by the spider sensory system. *Nature* **2014**, *516*, 222-226.
(50) Jung, H.; Park, C.; Lee, H.; Hong, S.; Kim, H.; Cho, S. J. Nano-cracked strain sensor with high sensitivity and linearity by controlling the crack arrangement. *Sensors (Switzerland)* **2019**, *19, 2834*.
(51) Lee, T.; Choi, Y. W.; Lee, G.; Kim, S. M.; Kang, D.; Choi, M. Crack-based strain sensor with diverse metal films by inserting an inter-layer. *RSC Advances* **2017**, *7*, 34810-34815.
(52) Tolvanen, J.; Hannu, J.; Jantunen, H. Stretchable and Washable Strain Sensor Based on Cracking Structure for Human Motion Monitoring. *Sci. Rep.* **2018**, *8*.
(53) Kim, T.; Lee, T.; Lee, G.; Choi, Y. W.; Kim, S. M.; Kang, D.; Choi, M. Polyimide encapsulation of spider-inspired crack-based sensors for durability improvement. *Applied Sciences (Switzerland)* **2018**, *8*, 367.
